# Electrostatic and Covalent Binding of an Antibacterial Polymer to Hydroxyapatite for Protection against *Escherichia coli* Colonization

**DOI:** 10.3390/ma16145045

**Published:** 2023-07-17

**Authors:** Sudip Chakraborty, Georgio Katsifis, Iman Roohani, Cyrille Boyer, David McKenzie, Mark D. P. Willcox, Renxun Chen, Naresh Kumar

**Affiliations:** 1 School of Chemistry, UNSW Sydney, Sydney, NSW 2052, Australia; 2 School of Physics, University of Sydney, Sydney, NSW 2006, Australia; 3 Charles Perkins Centre, University of Sydney, Sydney, NSW 2006, Australia; 4 School of Life and Environmental Sciences, University of Sydney, Sydney, NSW 2006, Australia; 5 School of Chemical Engineering, UNSW Sydney, Sydney, NSW 2052, Australia; 6 School of Optometry and Vision Science, UNSW Sydney, Sydney, NSW 2052, Australia

**Keywords:** antimicrobial peptides, antimicrobial polymers, biomaterials, hydroxyapatite, antimicrobial coatings

## Abstract

Orthopedic-device-related infections are notorious for causing physical and psychological trauma to patients suffering from them. Traditional methods of treating these infections have relied heavily on antibiotics and are becoming ineffectual due to the rise of antibiotic-resistant bacteria. Mimics of antimicrobial peptides have emerged as exciting alternatives due to their favorable antibacterial properties and lack of propensity for generating resistant bacteria. In this study, the efficacy of an antibacterial polymer as a coating material for hydroxyapatite and glass surfaces, two materials with wide ranging application in orthopedics and the biomedical sciences, is demonstrated. Both physical and covalent modes of attachment of the polymer to these materials were explored. Polymer attachment to the material surfaces was confirmed via X-ray photoelectron spectroscopy and contact angle measurements. The modified surfaces exhibited significant antibacterial activity against the Gram-negative bacterium *E. coli*, and the activity was retained for a prolonged period on the surfaces of the covalently modified materials.

## 1. Introduction

Orthopedic-device-related infections (ODRIs) pose serious technological and economic challenges to healthcare institutes and governments around the world, while causing physical and psychological trauma to patients suffering from them [1]. Although many of these infections are caused by Staphylococcus species [2], Gram-negative bacteria such as *E. coli* constitute a significant threat owing to the difficulty of treating these infections [3,4,5].

ODRIs have traditionally been treated with antibiotics [6,7,8], either as free forms or conjugated (electrostatic and covalent) to implants and with various modes of antibiotic release from implants [1,8,9]. However, a major drawback with these techniques is their over-reliance on antibiotics. Generous use of antibiotics has led to the emergence of antibiotic-resistant bacteria [10,11]. This has motivated scientists to explore alternative antibacterial agents. One class of such alternative antibacterial agents is antimicrobial peptides (AMPs) [12,13,14]. AMPs are usually amphiphilic, cationic peptides, some of which are known to act as components of the innate immune system in a variety of species [15,16,17,18]. They often exhibit broad-spectrum activity and are less susceptible to bacterial resistance, owing to their mode of action that involves non-specific binding to bacterial membrane surfaces, thereby causing membrane disruptions [19,20,21,22].

Their interesting properties notwithstanding, natural AMPs do not have ideal pharmacological properties [23,24,25]. Thus, synthetic mimics of AMPs have been explored [26,27,28]. These mimics incorporate the properties of AMPs that render them antibacterial, such as amphiphilicity and net positive charge [29]. However, unlike AMPs, the mimics are designed to be resistant to proteolytic cleavage, resistant to changes in pH and ionic conditions, and have higher solubility. A wide variety of molecules have been used as mimics. These include, but are not limited to, small peptides, oligopeptides, and non-peptide-based mimics [28,30,31]. Although the small molecule and peptide-based mimics have shown substantial promise, they can also suffer from certain limitations. These include poor biocompatibility, reduced in vivo activity, and often, poor activity against biofilms.

An exciting class of non-peptide-based AMP mimics is antimicrobial polymers (APs) [32,33,34]. APs are manufactured through controlled polymerization techniques and like other mimics of AMPs, emulate the physical properties of AMPs. Several papers have reported star-shaped polymers [35,36,37] and polymeric micelles [38] with antimicrobial activity that are also biocompatible, i.e., not cytotoxic towards mammalian cells. Cyrille et al. recently synthesized a library of single-chain polymeric nanoparticles (SCPNs) by systematically varying their chemical compositions [39,40,41]. These SCPNs folded into stable conformations in aqueous media via intramolecular hydrophobic interactions [39]. Changes in chemical composition were introduced deliberately to identify SCPNs with selectivity towards bacterial cells over mammalian ones, and to identify SCPNs with reduced susceptibility to bacterial resistance [40]. The SCPNs were active against both planktonic and biofilm bacteria and were also potent in dispersing biofilms [39]. Furthermore, the APs retained their activity in the presence of interfering serum proteins. Such APs represent promising alternatives to traditional antibiotics.

In the context of ODRIs, coating bone implant materials with antimicrobial agents that demonstrate a reduced susceptibility to bacterial resistance is crucial for the fight against antibiotic resistance, and to improve health outcomes of patients with implants. Hydroxyapatite (HA), a calcium phosphate-based bioceramic [42,43,44,45,46,47,48,49], is used widely in bone implant research, from classical implants [42,50,51] to tissue-engineered scaffolds [46,52], due to its chemical similarity to the inorganic mineral phase of natural bones and its excellent biocompatibility [53,54,55,56]. HA, therefore, serves as a suitable model material for testing the efficacy of antibacterial coatings for treating ODRIs. The two general strategies available for attaching antibacterial compounds to the surface of HA are covalent and physical (electrostatic) binding [57,58,59]. Both strategies have some advantages and some drawbacks associated with them. However, these depend on the specific details of the interactions between the antibacterial entity and the hydroxyapatite surface (and the bulk material as the case may be). The strength of attachment, the rate of release, etc., all play important roles in determining the effectiveness of a particular type of coating in combating bacterial growth. It is often not feasible to have a complete theoretical understanding of the factors that affect the performance of a particular coating. Therefore, it is crucial to explore multiple coating strategies experimentally in order to determine the optimal. We have previously demonstrated the efficacy of a small molecule peptidomimetic as a coating material for HA disc surfaces [60]. The HA surfaces were conjugated with the peptidomimetic via electrostatic bonding, and the coated material inhibited the growth of *S. aureus* [60].

The aim of the current study was to determine whether AP physically adsorbed or covalently bound to HA is able to influence the colonization by *E. coli*. For covalent attachment, an Plasma Immersion, Ion Implantation, and Deposition (PIIID) technique was used, followed by spin-coating. Attachment of the AP to HA was confirmed via XPS, and antibacterial activity was monitored via direct-contact antibacterial assay and SEM imaging. PIIID is a linker-free, single-step process for attaching compounds directly to surfaces of nonpolymeric materials [61,62,63,64]. PIIID has previously been used to attach AMPs to glass [61], and the attachment of the AMP to the PIIID-treated glass surface was confirmed via XPS [61]. In the current study, we used glass coverslips as control surfaces for comparison with this established protocol.

### Summary

In summary, ODRIs pose significant challenges to healthcare systems and patients. Unfortunately, the over-reliance on antibiotics for treatment has led to the emergence of antibiotic-resistant bacteria. As an alternative, antimicrobial peptides (AMPs) and their synthetic mimics have been explored due to their broad-spectrum activity and reduced susceptibility to bacterial resistance. Among the mimics, antimicrobial polymers (APs) show promise as they emulate the physical properties of AMPs and exhibit biocompatibility. Coating bone implant materials such as hydroxyapatite (HA) with antimicrobial agents is crucial in combating ODRIs. In this study, we report strategies for attaching an AP to HA surfaces and investigate the efficacy of such coated surfaces in combating colonization by the Gram-negative bacterium *E. coli*. The study aims to investigate the influence of mode of attachment of the AP (physically adsorbed or covalently bound) on the antibacterial activity of the coated surfaces.

## 2. Materials and Methods

### 2.1. Synthesis of HA Nanoparticles and HA Discs

HA nanoparticles and, subsequently, HA discs, were synthesized according to protocols described previously [60]. Briefly, ammonium phosphate dibasic (79.24 g) and calcium nitrate tetrahydrate (236.15 g) were weighed and added to two separate beakers that contained predefined volumes of MilliQ water (500 mL for ammonium phosphate dibasic and 1500 mL for calcium nitrate tetrahydrate). Ammonium phosphate dibasic was dissolved spontaneously in MilliQ water, while the calcium nitrate tetrahydrate solution containing the beaker was transferred to a hot plate and heated until it reached a temperature of 60 °C. Upon the latter reaching 60 °C, both the solutions were mixed. Mixing was carried out slowly in order to ensure that a pH of more than 7.5 was maintained during the entire process. The entire solution was passed through a filter paper after complete mixing was accomplished. The filtered cake containing the HA nanoparticles was then dried overnight at room temperature and the filtrate was discarded. The HA nanoparticles were then sintered for one hour at 1000 °C.

HA discs were fabricated using the sintered nanoparticles. Briefly, the nanoparticles were first disaggregated and powdered. Powder particles larger than 0.2 μm were separated out from the rest by running the powder through a sieve. Particles larger than 0.2 μm were ground further to bring their size down while the particles smaller than 0.2 μm were used to prepare a 40 wt% (40 g of nanoparticles in 60 g of water) mixture in water. The mixture was subsequently homogenized for 60 min inside a ball mill and then cast into cylindrical molds. The casts were dried overnight inside a vacuum desiccator. Dry, cylindrical HA discs were subsequently sintered for 6 h at 1100 °C inside a furnace.

### 2.2. Synthesis of PIIID-Modified HA Discs and Glass Surfaces

**Figure 1:**
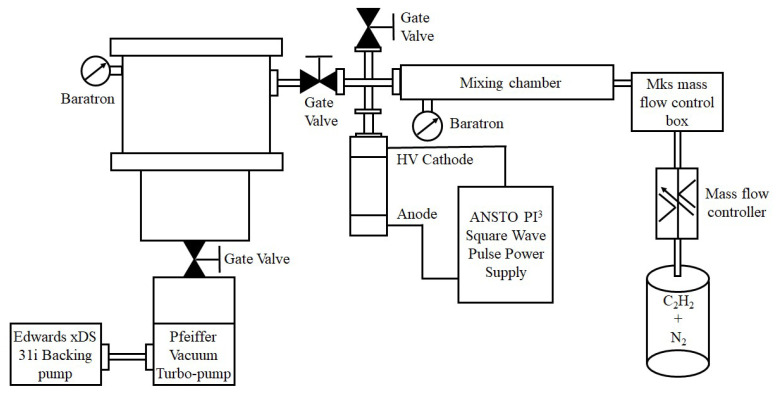
Schematic of the PIIID treatment process.

PIIID treatment of HA discs and glass surfaces was carried out using a dielectric barrier discharge (DBD) apparatus [65]. Nitrogen and acetylene gases were used at a pressure of 500 mTorr. The DBD was powered by a custom ANSTO switch-mode pulsed power supply with a square wave amplitude of −10 kV, pulse width of 40 µs, frequency of 500 Hz, and a pulse rise time of ~500 ns. The HA discs and glass cover slips were placed on the base of a borosilicate Erlenmeyer flask and treated on both sides for 5 min each. The chamber was evacuated to 10^−5^ torr to remove oxygen, before the treating gas was flowed in. Chamber evacuation was performed with a turbomolecular pump backed by a scroll pump and nitrogen and acetylene gases were flowed in equal volume ratios into the chamber at a combined pressure of 350 mTorr. A schematic of the PIIID treatment setup is presented in Figure 1.

### 2.3. Synthesis of Polymer-Coated HA Discs, PIIID-HA Discs and PIIID-Glass Surfaces

**Scheme 1:**
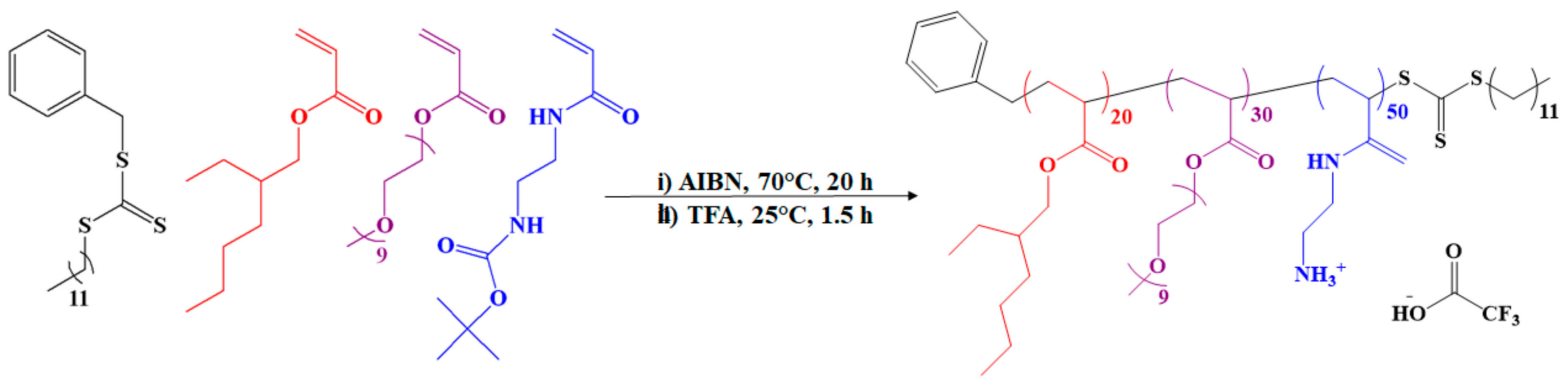
Synthesis of antimicrobial polymer (AP).

The AP used in this study was synthesized according to a protocol described by Boyer and coworkers [39,41]. It is an amphiphilic random ternary copolymer rationally designed to exhibit optimal antimicrobial activity and biocompatibility. The AP contains 20 repeat units of hydrophobic ethylhexyl group to aid in bacterial membrane disruption, 30 repeat units of biocompatible oligoethylene glycol with anti-fouling properties, and 50 repeat units of primary amino groups to aid in bacterial membrane attachment via electrostatic interactions. The AP was synthesized in large quantities via a controlled radical polymerization technique, known as reversible addition–fragmentation chain transfer (RAFT) polymerization [66,67]. A schematic of the synthesis, recreated from [41], is presented in Scheme 1. The reported minimum inhibitory concentration (MIC) of the AP against *E. coli* is 32 µg/mL, or 1.4 µM [39].

Native HA discs were conjugated to the polymer via physical interactions while PIIID-modified HA discs and glass surfaces were utilized for covalent conjugation with the AP. AP attachment to the HA discs, PIIID-HA discs, and PIIID-glass surfaces was achieved via spin coating. Briefly, 500 and 1000 μg of the polymer were dissolved in two different vials containing 1 mL of MilliQ water each, resulting in two solutions containing different concentrations of the polymer (500 μg/mL and 1000 μg/mL). Subsequently, HA discs, PIIID-HA discs and PIIID-glass coverslips were transferred to well plates and incubated in these solutions for 24 h at room temperature and under mild shaking. After incubation, the solutions were removed from the wells and the discs and coverslips were washed thrice with distilled water. The discs were then subjected to mild vortexing to ensure the removal of loosely bound and unbound polymer. After the washing and the vortexing steps, the discs and the coverslips were left to dry for 24 h at room temperature. Two different test groups (discs and coverslips coated with 500 and 1000 μg/mL) were used for all the experiments. Uncoated samples (incubated in MilliQ water without the polymer) were used as controls. HA discs incubated in 500 and 1000 μg/mL solutions of the polymer are referred to as HA 500p and HA 1000p, respectively, in the relevant places in this text, while the corresponding PIIID-treated discs and glass coverslips are referred to as PIIID-HA 500p, PIIID-HA 1000p, PIIID-glass coverslip 500p, and PIIID-glass coverslip 1000p, wherever necessary. To minimize confusion, the nature of the samples being discussed is specified in the text.

### 2.4. Characterization of PIIID-Modified and Polymer-Coated Samples

#### 2.4.1. Scanning Electron Microscopy (SEM) Imaging of PIIID-HA Discs and PIIID-Glass Coverslips

A field-emission scanning electron microscope (FE-SEM), NanoSEM 230, was used for all the imaging performed in this study. Native HA discs and PIIID-HA discs were imaged to observe the morphological changes brought about by the PIIID treatment on the surface of HA. For this, untreated and PIIID-HA discs were sputter coated with 30 nm of platinum in a Leica sputter coater, and then imaged under the microscope. Spot sizes between 3 and 5 and an accelerating voltage of 15 kV were found to be suitable for imaging.

#### 2.4.2. Fourier-Transform Infrared Spectroscopy (FTIR)

FT-IR spectroscopy was performed to analyze the surfaces of HA discs (untreated, PIIID-treated, and polymer-coated PIIID-treated). The motivation behind this analysis was to assess the types of chemical species introduced to the surface of native HA discs via the PIIID treatment. Furthermore, data from FT-IR and RAMAN spectroscopy are complementary in nature, and can therefore be used to corroborate each other. For this analysis, we used a Spectrum 100/Spotlight 400 FT-IR spectrometer and operated it within the scanning range of 650 to 4000 cm^−1^.

#### 2.4.3. RAMAN Spectroscopy

RAMAN spectroscopy was further performed to analyze the surfaces of untreated HA discs, PIIID-HA discs, and PIIID-HA discs coated with the two different concentrations of the polymer. Similar to FT-IR spectroscopy, the primary motivation behind this study was to assess the types of chemical species introduced to the HA surface via the PIIID treatment. Furthermore, RAMAN spectra of the PIIID-treated samples were used as an indicator of the success of the PIIID treatment. A grating of 1800 L/mm and a standard diode laser setting of 532 nm (green) was used for the analysis. In order to determine the extended spectra of the samples, the scanning range was adjusted from 44 to 4000 cm^−1^.

#### 2.4.4. X-ray Photoelectron Spectroscopy (XPS)

In order to qualitatively confirm the attachment of the polymer onto the unmodified HA discs, and the PIIID-HA discs, and PIIID-glass coverslips, and to further characterize the changes in the chemical composition of the HA disc surface brought about by the PIIID treatment, XPS was performed on the surfaces. For this analysis, a Thermo ESCALAB250Xi X-ray photoelectron spectrometer was used, and it was operated at a background vacuum greater than 2 × 10^9^ mbar. A mono-chromated Al Kα (energy 1486.68 eV) was used as the X-ray source and a spot size of 500 μm was found to be suitable for the analysis. The device was operated at a power of 120 W and at a photoelectron take-off angle of 90°.

To characterize the nature of the attachment of the polymer to the PIIID-HA discs and PIIID-glass, these surfaces, along with the corresponding polymer-coated ones, were incubated in a solution of 2% sodium dodecyl sulfate (SDS) for one hour at a temperature of 70 °C. This assay was performed in a water bath to ensure a constant temperature throughout the one-hour period. SDS wash at an elevated temperature is a standard procedure for the removal of electrostatically bound entities from treated surfaces [61,62]. Subsequently, the samples were washed thrice with MilliQ water. After the final wash, the discs and coverslips were left to dry at room temperature for 24 h, and then analyzed via XPS. The elemental composition of PIIID-treated and polymer-coated surfaces before and after SDS treatment were analyzed and compared to that of the uncoated and PIIID-only-treated surfaces. This assay was performed as a qualitative test for the covalency of the bonds between the polymer and the PIIID-modified surfaces and was not performed on the samples that were used in other physicochemical and biological testing.

#### 2.4.5. Contact Angle Measurement

The effect of PIIID treatment and subsequent polymer attachment on the wettability of glass coverslip surface was evaluated via contact angle measurement. The analysis was performed only on the glass coverslips as the HA discs did not allow for the formation of stable droplets due to their extremely porous nature. However, the change, or lack thereof, of the wettability the glass surface due to PIIID treatment and subsequent polymer attachment can serve as a reasonable indicator of what happens to HA surfaces too after similar modifications. For this analysis, a ramé-hart contact angle goniometer was used. A syringe was used to deposit water droplets on the surfaces of glass coverslips that were placed on a stage. The volume of the droplets deposited on the samples was kept approximately the same. Three droplets were deposited per sample and the contact angle was measured using the software DROPimage Standard (v2.1.3).

### 2.5. Antibacterial Activity of the Modified Surfaces

*E. coli* K12 strain (ATCC 10798; isolated from feces of a diphtheria convalescent) was used to assess the antibacterial performance of the surfaces described in this study. The antibacterial activity of the unmodified and PIIID-treated HA discs coated with the polymer, and PIIID-glass surfaces coated with the polymer was determined by culturing *E. coli* (ATCC 10798) directly on them [68]. Surfaces devoid of the polymer were used as controls.

#### 2.5.1. Direct Contact Antibacterial Assay

In the first study, the number of bacteria growing in the solution in contact with the polymer-coated surfaces was determined. Briefly, *E. coli* K12 (ATCC 10798) was grown in Mueller Hinton Broth at 37 °C overnight. The next day, the bacteria were collected via centrifugation for 10 min at 3500 rpm. Subsequently, the bacteria were dissolved in fresh broth to an absorbance of 0.1 at 600 nm, corresponding to a bacterial concentration of 1 × 10^8^ bacteria/mL. The broth was further diluted 1000-fold to obtain a final bacterial concentration of 10^5^ bacteria/mL.

Three sets of samples were used in this study, and three groups were included in each set. The first set comprised untreated HA disc as control, and HA disc incubated in 500 and 1000 μg/mL polymer as the two test groups. The second set comprised PIIID-glass coverslip as the control group, and PIIID-glass coverslips incubated in 500 and 1000 μg/mL polymer as the two test groups. Finally, the third set comprised PIIID-HA discs as the control group, and PIIID-HA discs incubated in 500 and 1000 μg/mL polymer as the two test groups. Samples were transferred to 96-well plates and incubated overnight at 37 °C in MHB media containing 10^5^ bacteria/mL. Post incubation, the plates were retrieved and the media from each well was collected into separate Eppendorf tubes and serially diluted from 1 to 10-fold. Sterile PBS was used for preparing the dilutions. The diluted medium was subsequently collected from the tubes and pipetted out onto nutrient agar plates. For this, nutrient agar plates were divided into five segments of roughly equal area, and each segment was used for growing bacteria corresponding to a single dilution. Therefore, ten different segments (two nutrient agar plates) were utilized to grow bacteria corresponding to all the ten dilutions of a single group. The plates containing the diluted media were further incubated overnight at 37 °C. Post incubation, the number of visible single bacterial colonies, referred to as the Colony Forming Units (or CFUs), in each segment of the nutrient agar plates were counted, and the values were assigned to the corresponding dilutions. The CFUs in nth dilution was then used to calculate the CFUs/mL of the undiluted media using the following formula: $\frac{\mathrm{CFUs}}{\mathrm{mL}}\;\left(\mathrm{undiluted}\;\mathrm{media}\right)=\mathrm{CFUs}\;\mathrm{in}\;\mathrm{nth}\;\mathrm{dilution}\;\times 10^{n}$.

#### 2.5.2. Bacterial Adhesion Assay

A modification of a previous protocol was followed to quantify the number of bacteria attached to surfaces of the materials [69]. Three sets of samples were used in this study, and three groups were included in each set. The first set comprised untreated HA discs as control and HA discs incubated in 500 and 1000 μg/mL polymer as the two test groups. The second set comprised PIIID-glass coverslips as the control group and PIIID-glass coverslips incubated in 500 and 1000 μg/mL polymer as the two test groups. Finally, the third set comprised PIIID-HA discs as the control group and PIIID-HA discs incubated in 500 and 1000 μg/mL polymer as the two test groups.

Briefly, the samples were transferred to 96-well plates and incubated overnight at 37 °C in MHB media containing 10^5^ bacteria/mL. Post incubation, the samples were washed three times with PBS with mild shaking for 5 min in order to remove loosely attached bacteria. All the samples barring the glass coverslips were subsequently removed from the wells and transferred to Eppendorf tubes containing broth. The tubes were then placed on a vortex mixer and the samples were vortexed vigorously for 5 min. The glass coverslips were retained in the well plates and submerged in fresh broth. The plates were placed on a vortex mixer and the coverslips were vortexed gently for 5 min. Vortexing was performed to ensure the dissociation of strongly attached bacteria from the discs and the coverslips. After vortexing, the media from the Eppendorf tubes (in case of the HA disc) and the well plates (in case of the glass coverslips) were collected into separate Eppendorf tubes and serially diluted from 1- to 10-fold. Sterile PBS was used for preparing the dilutions. The diluted medium was subsequently collected from the tubes and pipetted out on nutrient agar plates. For this, the nutrient agar plates were divided into five segments of roughly equal area, and each segment was used for growing bacteria corresponding to a single dilution. Therefore, ten different segments (two nutrient agar plates) were utilized to grow bacteria corresponding to all the ten dilutions of a single group. The plates containing the diluted media were further incubated overnight at 37 °C. Post incubation, the number of visible single bacterial colonies, referred to as colony forming units (or CFUs), in each segment of the nutrient agar plates were counted, and the values were assigned to the corresponding dilutions. The number of CFUs in the nth dilution was then used to calculate the CFUs/mL of the undiluted media using the following formula: $\frac{\mathrm{CFUs}}{\mathrm{mL}}\;\left(\mathrm{undiluted}\;\mathrm{media}\right)=\mathrm{CFUs}\;\mathrm{in}\;\mathrm{nth}\;\mathrm{dilution}\;\times 10^{n}$.

#### 2.5.3. SEM Imaging of Bacteria on the Surfaces

Bacteria attached to the surfaces were also imaged using SEM. Untreated HA discs and HA discs incubated in 500 and 1000 µg/mL polymer, PIIID-HA discs and PIIID-HA discs incubated in 500 and 1000 µg/mL polymer, and PIIID-glass coverslips and PIIID-glass coverslips incubated in 500 and 1000 µg/mL polymer were imaged in this study. Briefly, the samples were transferred to 96-well plates and incubated overnight at 37 °C in MHB media containing 10^5^ bacteria/mL. Post incubation, the samples were washed three times with PBS under mild shaking condition for 5 min in order to get rid of loosely attached bacteria. Subsequently, the firmly attached bacteria were fixed to the surfaces via incubation for two hours in a 2.5% glutaraldehyde solution at ambient temperature. Post fixing, the samples were washed sequentially with increasing concentrations of ethanol, starting at 50%, then 60%, then 70%, all the way up to 100% ethanol. After the last wash, the samples were dried at the critical point of carbon dioxide in a critical point dryer and then sputter coated with 30 mm of platinum in a Leica sputter coater. For native HA discs (uncoated and polymer-coated), a spot size of 3 and an accelerating voltage of 15 kV were found to be suitable for imaging. For PIIID-glass surfaces and PIIID-HA discs, a spot size of 3 and an accelerating voltage of 5 kV were found to be suitable for imaging.

### 2.6. Analysis of Retention of Antibacterial Activity

#### 2.6.1. XPS Analysis of Surface Elemental Composition before and after Release of Polymer

The surfaces of HA and PIIID-HA discs coated with 1000 µg of the antimicrobial polymer (HA + 1000p, PIIID-HA + 1000p) were analyzed via XPS after their incubation in PBS for 7 days. A Thermo ESCALAB250Xi X-ray photoelectron spectrometer was used at a background vacuum higher than 2 × 10^9^ mbar. A mono-chromated Al Kα (energy 1486.68 eV) was used as the X-ray source and the power was maintained at 120 W. A photoelectron take-off angle of 90° and a spot size of 500 μm was chosen for the analysis.

#### 2.6.2. Antibacterial Assay

The ability of the modified HA discs to retain their antibacterial activity over a period of two weeks in PBS was tested. Newly coated HA discs (both physically and covalently coated) were incubated in PBS inside 24-well plates. Each day after incubation, the PBS from each sample was removed and replaced with fresh PBS. After 7 and 14 days of incubation, the samples were removed and air dried. The dry samples were then incubated with fresh bacteria at a concentration of 10^5^ bacterial/mL of media. Subsequently, after 24 h of bacterial growth, the total number of bacteria growing in the supernatant and on the surface of the discs was measured according to the protocols already described above. The degree of reduction in the number of viable bacteria, both in the supernatant and the sample surface, was compared to the antibacterial activity of newly coated discs. 

### 2.7. Anti-Biofilm Activity

#### 2.7.1. In Vitro Inhibition of Biofilm Formation Assay

The ability of AP-coated HA discs and glass coverslips to inhibit formation of biofilms was measured by quantifying the biomass of biofilms via a crystal violet staining assay. Briefly, *E. coli* K12 (ATCC 10798) bacteria were grown in MHB for 18 h and then diluted in MHB to obtain a final bacterial concentration of 10^6^ CFU/mL. 100 µL of the bacterial solution was then added to the wells of a 96-well plate containing HA, PIIID-HA, HA + 1000p, and PIIID-HA + 1000p discs and glass, PIIID-glass, glass + 1000p, and PIIID-glass + 1000p coverslips. The plates were subsequently incubated at 37 °C for 48 h. Following incubation, the media from the wells were pipetted out and the discs were washed thrice with 125 µL of Milli-Q water to get rid of loosely bound bacteria and residual polymer. The adherent biofilms on the discs were then stained with 125 µL of 0.1% (*w*/*v*) crystal violet solution at room temperature. After 10 min of staining, the discs were washed again, being washed thrice with 200 µL of Milli-Q water. After the final wash, 200 µL of 70% (*w*/*v*) ethanol was added to each well to solubilize the crystal violet. The ethanol–crystal violet solutions from each well were then transferred to another 96-well plate and their optical densities were measured at 595 nm in a microplate reader. The percentage of biofilm inhibition compared to negative controls (HA discs and glass coverslip, respectively) was calculated using the following formula.

$$Reduction\;in\;biomass\;\left(\%\right)=\frac{OD_{negative\;control}-OD_{sample}}{OD_{negative\;control}}\times 100.$$

#### 2.7.2. SEM Imaging of Biofilm Bacteria on the Surfaces

In addition to quantifying the biofilm biomass, bacteria attached to the surfaces were also imaged under SEM. Briefly, the samples were transferred to 96-well plates and incubated overnight at 37 °C in MHB media containing 10^5^ bacteria/mL. Post incubation, the plates were retrieved, and the medium from each well was discarded by pipetting. The samples were washed thrice with PBS under mild shaking conditions for 5 min in order to get rid of loosely attached bacteria. Subsequently, the strongly attached bacteria were fixed to the surfaces via incubation for two hours in a 2.5% glutaraldehyde solution at room temperature. Post fixing, the samples were washed sequentially with increasing concentrations of ethanol, starting at 50%, then 60%, then 70%, all the way up to 100% ethanol. After the last ethanol wash, the samples were dried at the critical point of carbon dioxide in a critical point dryer, sputter coated with 30 mm of platinum in a Leica sputter coater and taken for imaging. For native HA discs (uncoated and polymer-coated), a spot size of 3 and an accelerating voltage of 15 kV were found to be suitable for imaging. A spot size of 3 and an accelerating voltage of 5 kV were found to be suitable for imaging all the samples.

### 2.8. Assessment of Cytotoxicity

#### 2.8.1. Cell Culture

In vitro cytotoxicity studies were performed to analyze the biocompatibility of the modified surfaces. Human fetal osteoblast cells, hFOB 1.19 (at passage 4), were revived from a frozen stock in DMEM/F-12 media supplemented with 10% Fetal Bovine Serum (FBS), streptomycin (5000 μg/mL), and penicillin (5000 unit/mL). The cells were then grown in a humidified CO_2_ chamber which was maintained at 5% CO_2_ and 37 °C. After one passage, the cells were used for alamar blue cytotoxicity assay.

#### 2.8.2. Alamar Blue Assay

The cytotoxicity of the physically and covalently coated HA disc surfaces was determined by measuring the metabolic activity of hFOB 1.19 cells grown on the discs for 24 and 72 h. For the physically coated discs, uncoated HA discs were used as the control. For the covalently coated PIIID-modified discs, uncoated PIIID-HA discs were used as the control. The cytotoxicity of all the groups were compared to that of nascent tissue culture plates. Briefly, the discs were incubated in DMEM/F-12 media supplemented with 10% FBS for 24 h to ensure the adsorption of nutrients from the media onto the surface of the materials. The medium was discarded, and the discs were resuspended in fresh media for cell seeding. Cells harvested by trypsinized from tissue culture plates were seeded onto the discs at a density of 10^4^ cells/disc and cultured up to 72 h. The medium was replaced every 24 h. After 24 and 72 h of cell growth, the medium was supplemented with alamar blue reagent at 10% (*v*/*v*). The cells were then incubated for 2 h in a humidified CO_2_ chamber at 37 °C. Post incubation, the medium was pipetted out from the wells and transferred to a 96-well microtiter plate. The absorbance of the medium was then measured at 570 nm with a reference wavelength of 600 nm. The cell viability was compared to cells grown on tissue culture plates. The percentage cell viability was then calculated from the absorbance values using the following formula:

$$\mathrm{Percentage}\;\mathrm{cell}\;\mathrm{viability}=\frac{\left(M2\times A1\right)-\left(M1\times A2\right)}{\left(M2\times P1\right)-\left(O1\times P2\right)}\times 100,$$

where:

M1 = molar extinction coefficient (E) of oxidized alamar blue (blue) at 570 nm;

M2 = molar extinction coefficient (E) of reduced alamar blue (red) at 600 nm;

A1 = absorbance of test wells at 570 nm;

A2 = absorbance of test wells at 600 nm;

P1 = absorbance of positive growth control well (cells in tissue culture plate) at 570 nm;

P2 = absorbance of positive growth control well at 600 nm.

### 2.9. Statistics

All data presented in this study are plotted as mean ± standard deviation. Unless mentioned otherwise, all the experiments were performed in triplicate and repeated at least three times. GraphPad Prism software (10.0.0) was used to statistically analyze the data from the experiments. One-way Analysis of Variance (one-way ANOVA; GraphPad Prism) was used to determine significant differences between groups and *p*-values less than 0.05 were considered statistically significant. The following symbols are used to signify statistical significance throughout the article: ns = no significance (*p* > 0.05); * = *p* < 0.05; ** = *p* < 0.01; *** = *p* < 0.001; **** = *p* < 0.0001.

## 3. Results

### 3.1. Synthesis of PIIID-HA Discs and PIIID-Glass

**Figure 2:**
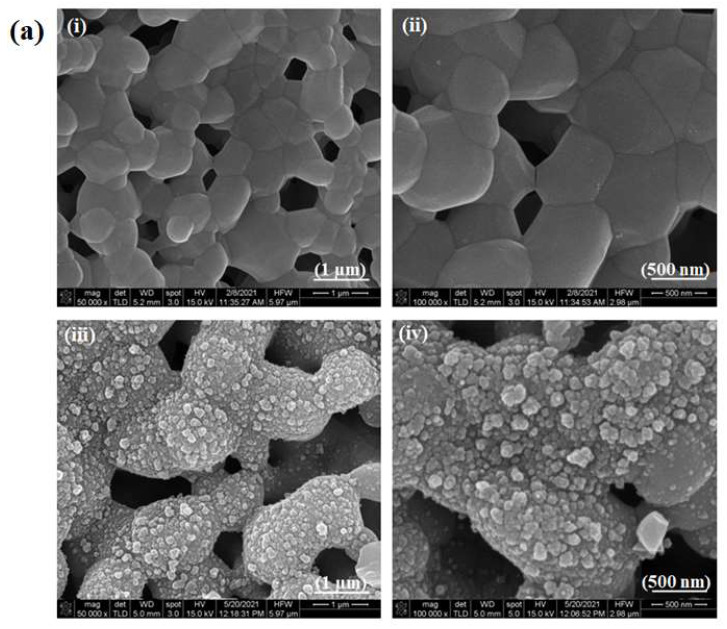

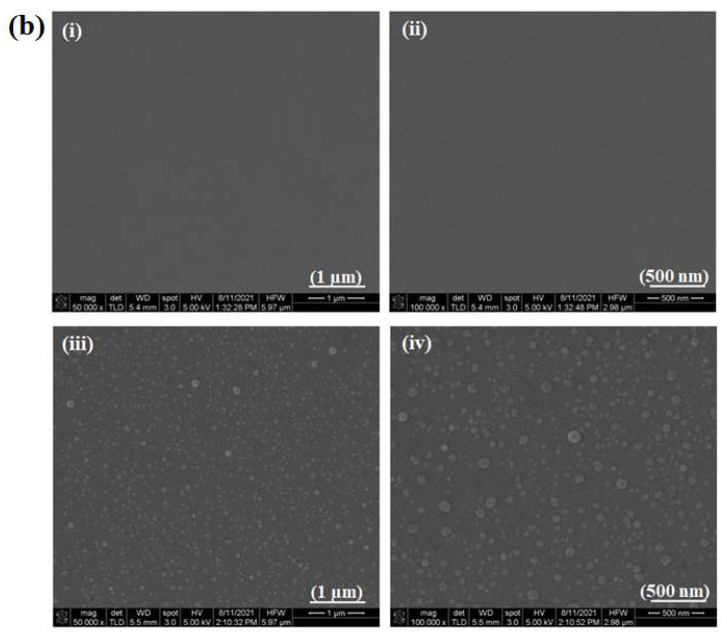
Scanning electron micrographs of HA disc surface (**ai**,**aii**) and PIIID-HA disc surface (**aiii**,**aiv**). Micrographs of glass coverslip (**bi**,**bii**), and PIIID-glass coverslip (**biii**,**biv**).

The surface morphologies of the HA discs, PIIID-HA discs, glass coverslips, and PIIID-glass coverslips were visualized via SEM. The PIIID process deposits a layer of reactive polymer over the surface on which it is performed. Therefore, our starting point was to observe the morphological changes brought about by this technique on HA and glass surfaces. The surface of the PIIID-HA discs exhibited a blistered appearance, while no such feature was observed on the unmodified HA disc surface (Figure 2a). Similarly, a blistered appearance was also observed for the PIIID-glass surface while no such feature was observed on unmodified glass surface (Figure 2b). The modified features on the surfaces of PIIID-HA discs and PIIID-glass are a result of the PIIID treatment.

### 3.2. Synthesis and Characterization of Polymer-Coated HA Discs, PIIID-Glass and HA Discs

#### 3.2.1. Fourier-Transform Infrared Spectroscopy (FTIR)

**Figure 3:**
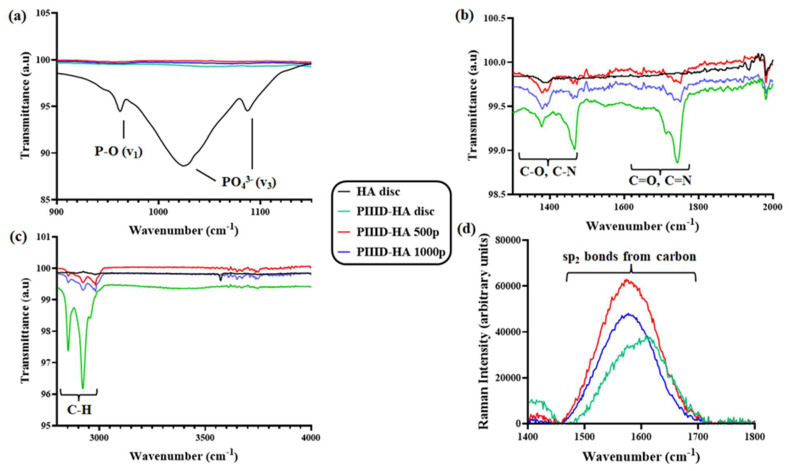
FT-IR spectra of the HA disc, PIIID-HA disc, PIIID-HA 500p, and PIIID-HA 1000p spanning three regions: (**a**) 900–1200 cm^−1^, (**b**) 1300–2000 cm^−1^, and (**c**) 2800–4000 cm^−1^. (**d**) RAMAN spectra of the HA disc, PIIID-HA disc, PIIID-HA 500p, and PIIID-HA 1000p spanning 1400–1800 cm^−1^.

The chemical groups present on the surfaces of unmodified and modified HA discs were analyzed via FT-IT spectroscopy. The FT-IR spectra of untreated HA discs revealed peaks corresponding to the functional groups present in pristine HA (Figure 3a). A peak around 960 cm^−1^, corresponding to v_1_ nondegenerate symmetric stretching characteristic of P-O bonds was observed in the spectra of HA. The spectra of HA also revealed peaks associated with the v_3_ vibration mode of the PO_4_^3−^ at 1036 and 1095 cm^−1^. The FT-IR spectrum of the PIIID-HA discs showed the peaks corresponding to the groups introduced by the PIIID treatment, and masked the peaks found in pure HA. Peaks were observed between 1650 and 1800 cm^−1^ (Figure 3b). These peaks correspond to C=O and C=N groups introduced to the surface via the PIIID treatment [70]. Peaks corresponding to C-O and C-N groups were observed between 1150 and 1400 cm^−1^. Peaks were also observed between 2800 and 3000 cm^−1^ corresponding to the C-H bonds present in acetylene (Figure 3c). Attachment of the polymer led to a reduction in the intensity of the peaks while no changes were observed in their locations. The complete FT-IR spectra of all the samples spanning the entire range of detection (45–4000 cm^−1^) are provided in Figure S1a.

#### 3.2.2. RAMAN Spectroscopy

The surfaces of the PIIID-HA discs, PIIID-HA 500p, and PIIID-HA 1000p were further analyzed via RAMAN spectroscopy. The RAMAN spectra of nascent HA discs displayed the characteristic peaks of HA. Peaks were observed at 1076 cm^−1^, 1046 cm^−1^, 1030 cm^−1^, and 961 cm^−1^, corresponding to the asymmetric and symmetric stretching modes of the PO_4_ group (Figure S1c). Peaks corresponding to the triply degenerated bending mode of the O-P-O bond were observed around 610 cm^−1^, 594 cm^−1^, and 582 cm^−1^ (Figure S1c). Finally, peaks corresponding to the doubly degenerated mode of the same bond were observed at 433 cm^−1^ and 447 cm^−1^ (Figure S1c). PIIID-HA discs and the discs coated with the polymer exhibited these peaks too.

The RAMAN spectra of PIIID-HA discs further revealed the characteristic peaks corresponding to the groups added by the PIIID treatment. Notably, peaks were observed between the wavenumbers 1500 and 1700 cm^−1^ (Figure 3d). These peaks correspond to sp^2^ bonds present in the carbon matrix of the film deposited via the PIIID treatment [70]. Native HA discs did not display any peaks between 1500 and 1700 cm^−1^, and therefore, their spectra are not visible in the graph in the figure. The complete RAMAN spectra of all the samples spanning the entire range of detection (50–1800 cm^−1^) are provided in Figure S1b.

#### 3.2.3. X-ray Photoelectron Spectroscopy (XPS)

Attachment of the polymer to the surfaces of HA discs, PIIID-HA discs, and PIIID-glass coverslips was confirmed via XPS. A significant increase in the percentage of the surface occupied by carbon and nitrogen was observed on HA discs that were physically bonded to the polymer (Table 1). Untreaded HA discs had a surface carbon content of 18.2% and a negligible amount of nitrogen (0.1%). The carbon on the surface of untreated HA discs can be attributed to contaminants and gases present in the atmosphere that get adsorbed to the surfaces of materials during their preparation, transport, etc. The most common species contributed by the contaminants is COOH. Similarly, the negligible amount of nitrogen on the surface can also be attributed to environmental contaminants. The surface of HA discs incubated in 500 and 1000 µg/mL polymer solutions exhibited carbon contents of 28.9 and 26.9%, respectively. The increase in the percentage of surface carbon is a consequence of the adsorption of the polymer on these surfaces. This conclusion is further corroborated by the significant increase in the percentage of surface nitrogen, which went up to 3.8 and 3.4% for the respective samples. There was also a decrease in the percentage of surface oxygen with the attachment of the polymer. Untreated HA discs exhibited a surface oxygen of 51.1%, while the values were 43.1 and 44.6% for HA discs incubated in 500 and 1000 µg/mL polymer, respectively.

PIIID treatment results in the deposition of a thin carbon film containing reactive radicals and functional groups on the HA disc surface [70]. The identities of some of these groups were identified via FT-IR and RAMAN spectroscopy as described above, and the results were corroborated during XPS analysis. An increase in the surface carbon and nitrogen percentages was observed (Table 1) on HA surface after PIIID treatment. Surface carbon on the HA disc after PIIID treatment was 66.4%, up from just 18.2% in the untreated HA disc. In-depth analysis of the types of carbon species present on the surface revealed the predominance of C-O, C-H, C-N, and C=N groups. Similarly, surface nitrogen went up from 0.1% to 23.1% after PIIID treatment. Most of the extra nitrogen was contributed by N-C and N=C species. However, there was a big drop in the percentage of surface oxygen, from 51.1% in untreated HA disc, to 9.3% in PIIID-HA disc. This can be attributed to the masking of the oxygen present in the underlying HA in the form of phosphates. The predominant species of oxygen on HA surface after PIIID treatment were found to be O-C, while some amount of O=C was also detected. Upon the attachment of the polymer, an increase in the amount of oxygen was observed. PIIID-HA discs incubated in 500 µg/mL polymer exhibited a surface oxygen content of 14.8%, while PIIID-HA discs incubated in 1000 µg/mL polymer exhibited a surface oxygen content of 14.3%. All the extra oxygen was found to be either in the O-C or in the O=C forms, more of which was introduced to the surface due to the attachment of the polymer. There was a reduction in the surface nitrogen content, with PIIID-HA discs incubated in 500 and 1000 µg/mL of polymer exhibiting 18.2 and 17.8% surface nitrogen, respectively. However, unlike the surface of PIIID-HA discs, most of the nitrogen on these polymer-coated surfaces was identified as belonging to the N-H species, and a reduced amount was identified as belonging to the N-C species, indicating the attachment of the polymer. PIIID treatment also uniformly covered the underlying HA structure as evidenced by the negligible amounts of calcium (0.6%) and phosphorus (0.4%) found on the PIIID-HA discs, amounts which were significantly lower than those found on untreated HA discs (calcium 17.8% and phosphorus 10%).

XPS data from the glass coverslips revealed similar trends in the amount of carbon, nitrogen, and oxygen as the HA discs (Table 2). PIIID treatment increased the surface carbon and nitrogen content from 21.2% and 0% on untreated glass to 73.6% and 12.3% on PIIID-glass coverslips. The predominant contributors to the increased carbon content were found to be the C-O, C-H, C-N, and C=N species, whereas the primary contributors to the increased nitrogen content were found to be the N-C and N=C species. The oxygen content on the surface decreased from 55.4% on untreated glass to 12.3% on PIIID-glass coverslips. The attachment of the polymer reduced the surface carbon content to 58.7% and 59.7% on PIIID-glass incubated in 500 and 1000 µg/mL polymer solutions, and the nitrogen content was reduced to 7.1% and 6.1% on the respective samples. However, the primary contributor to the surface nitrogen was found to be the N-H species. Like PIIID-HA discs, there was a decrease in the surface oxygen content from 55.4% on untreated glass to 12.3% on PIIID-glass. Furthermore, there was an increase in the surface oxygen content from 12.3% on PIIID-glass coverslips to 19.7% and 20% on PIIID-glass incubated in 500 and 1000 µg/mL polymer solutions, respectively (Table 2). O-C and O=C were the primary contributing species of oxygen on these surfaces, and they were contributed by the attached polymer.

**Figure 4:**
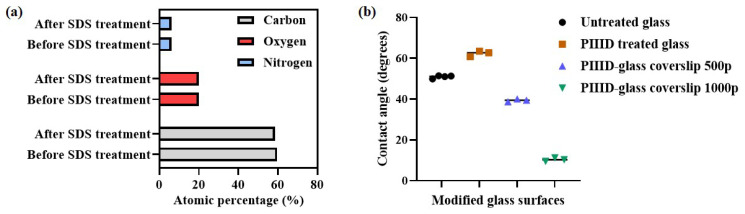
(**a**) Carbon, oxygen, and nitrogen content on PIIID-glass surfaces determined via XPS before and after treatment with SDS; (**b**) water–glass contact angle values on various glass surfaces.

**Figure 5:**
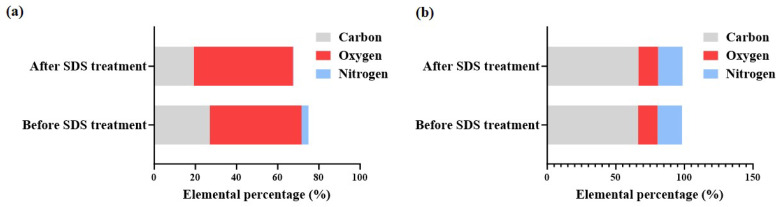
(**a**) Comparison of carbon, oxygen, and nitrogen content on HA 1000p surface before and after SDS treatment; (**b**) comparison of carbon, oxygen, and nitrogen content on PIIID-HA 1000p surface before and after SDS treatment.

The covalent bonds formed between the polymer and the PIIID-HA (Table 3) and glass surfaces (Figure 4a) was determined via XPS after the polymer-bound surfaces were subjected to washing in SDS, which has been shown to remove tightly adsorbed compounds [61,62]. The elemental composition of the PIIID-HA surfaces (uncoated and coated with the polymer) did not change significantly after the SDS treatment, suggesting that SDS did not have a significant impact on the surface composition of the discs (Figure 5b). Conversely, HA discs coated with the polymer did exhibit a significant change in surface elemental composition upon SDS treatment (Figure 5a). The XPS data indicate that the polymer was primarily bound covalently to the surfaces of the PIIID-HA discs. However, it should be noted that XPS reveals the composition of only the surface of a material up to a limited thickness. Since the HA discs used in this study were three dimensional and possessed a porous architecture, we cannot rule out the possibility of some of the polymer being physically entrapped inside the pores of the PIIID-HA discs at depths exceeding the limits of the PIIID treatment and of the XPS instrument.

#### 3.2.4. Contact Angle Measurement

Attachment of the polymer to PIIID-glass surfaces was further confirmed by measuring the changes in the hydrophobicity of the polymer-coated surfaces compared to an uncoated control surface. Furthermore, the change in the surface hydrophobicity brought about by PIIID treatment of native glass coverslips was also observed. PIIID modification resulted in an increase in the hydrophobicity of the surface (Figure 4b), raising the contact angle from 51 ± 1° (unmodified control glass surface) to 62 ± 1° (PIIID-glass surface). PIIID-glass incubated in polymer solutions of concentrations 500 μg/mL and 1000 μg/mL exhibited contact angles of 39 ± 1° and 10 ± 1°, respectively. Thus, a dose-dependent increase in the hydrophilicity of the surface was observed in response to attachment of the polymer. Although it was not possible to perform the same study on PIIID-HA surfaces owing to their porous nature, it can reasonably be concluded that the surface of HA is similarly modified upon PIIID treatment and subsequent polymer attachment. This is due to the fact that the PIIID treatment deposits a layer of reactive entities on the HA and glass surface which is at least 10 nm in thickness, and therefore, the surface hydrophobicity is primarily a function of the plasma coated layer and the added polymers, not the HA or glass underneath [63,64]. The negligible amount of calcium and phosphorus signals in the XPS spectra of PIIID-HA discs and the complete lack of silicon signal in the XPS spectra of PIIID-glass coverslips point to a layer thickness of at least 10 nm (the resolution of XPS) [71].

### 3.3. Antibacterial Activity

**Figure 6:**
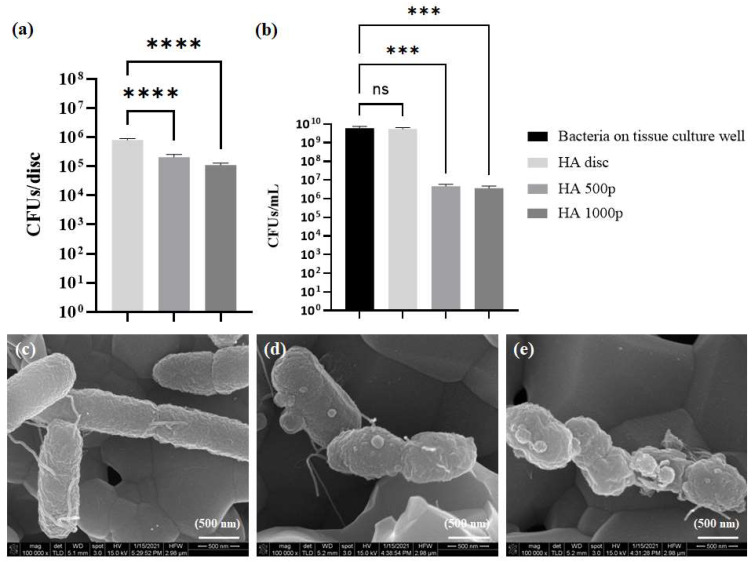
Antibacterial activity of HA discs physically coated with the polymer. (**a**) Antibacterial activity on coated disc surfaces, (**b**) activity in the surrounding media. Scanning electron micrographs of *E. coli* growing on (**c**) untreated HA disc, (**d**) HA 500p, and (**e**) HA 1000p. ns = no significance, *** = *p* < 0.001; **** = *p* < 0.0001.

Two distinct antibacterial parameters were quantified to assess the antibacterial performance of freshly coated samples. First, the number of bacteria growing in the supernatant surrounding the coated samples was quantified, and then, the number of bacteria growing attached to the surface of the samples was quantified. The HA discs that were physically conjugated to the polymer demonstrated higher antibacterial activity in the supernatant than the uncoated control discs, with both the discs (incubated in 500 and 1000 µg/mL polymer, respectively) exhibiting a 3-log_10_ reduction in the number of viable bacteria in the media (Figure 6b). No dose-dependent increase in the antibacterial activity was observed, as both the concentrations of the polymer yielded discs of similar antibacterial activity. This could be due to quite a few factors, including a saturation of the polymer on the HA surface, and a similar release kinetics of the polymer from the discs. However, these hypotheses need to be tested experimentally.

Similarly, the number of bacteria attached to the polymer-coated discs after one day of growth was lower compared to the control HA disc (Figure 6). However, unlike the bacteria in the supernatant, there was only a one-log_10_ reduction in the number of viable bacteria on both the coated discs. Additionally, unlike the bacteria in the supernatant, a dose-dependent increase in the toxicity towards bacteria on the surface was observed.

The morphology of the bacteria attached to disc surfaces was further observed under SEM. A reduced number of bacteria was observed on the surface of the polymer-coated discs, which was in accordance with the quantitative assay described above. Furthermore, *E. coli.* K12 exhibited a cylindrical rod-like morphology on all the discs, and no structural deformities were visible on the bacteria grown on the uncoated disc (Figure 6c). However, blebbing of the cell membrane was visible on the bacteria grown on HA disc incubated in 500 µg/mL polymer (Figure 6d). The blebbing was even more pronounced in the membrane of bacteria grown on the HA disc that was incubated in 1000 µg/mL polymer (Figure 6e).

**Figure 7:**
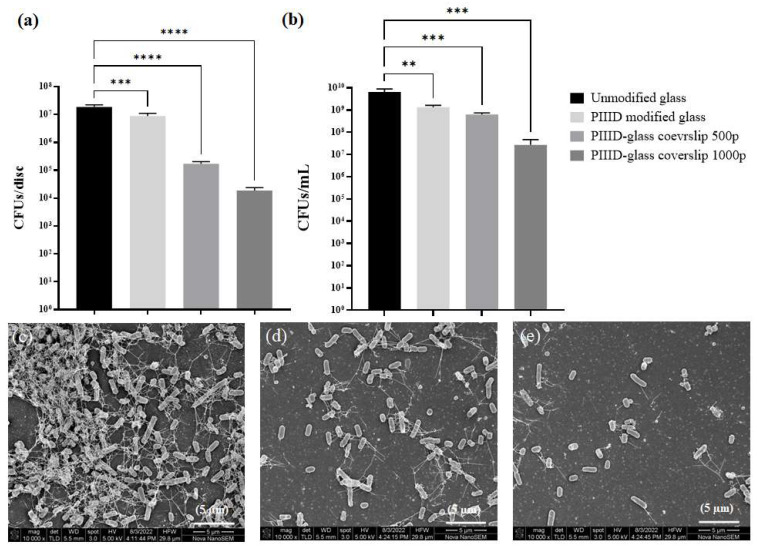
Antibacterial activity of glass coverslips covalently coated with the polymer. (**a**) Antibacterial activity on coated glass surface and (**b**) activity in the surrounding media. Scanning electron micrographs of *E. coli* growing on (**c**) untreated glass coverslip, (**d**) PIIID-glass coverslip 500p, and (**e**) PIIID-glass coverslip 1000p. ** = *p* < 0.01; *** = *p* < 0.001; **** = *p* < 0.0001.

The antibacterial activity of PIIID-glass surfaces coated with the polymer was analyzed in order to evaluate the effect of covalent binding on the activity of the polymer. AMPs have previously been attached to glass via PIIID and maintained their antimicrobial activity [61]. Similar results were observed with our polymer. PIIID modification of the glass surface imparted some antibacterial property to it, and it resulted in a 0.5-log_10_ reduction in the number of viable bacteria growing in the supernatant (Figure 7b). Attachment of the polymer led to further increase in the antibacterial activity, with PIIID-glass incubated in 500 µg/mL polymer exhibiting a one-log_10_ reduction in the number of viable bacteria compared to the control, and PIIID-glass incubated in 1000 µg/mL polymer exhibiting a 2.5-log_10_ reduction in the number of viable bacteria compared to the control (Figure 7b). The detection of antibacterial activity in the supernatant poses some interesting questions. Unlike physically attached polymers, covalently bound polymers aren’t expected to leach out into the surrounding solution within one day of incubation. However, it is quite possible that some of the polymer is entrapped in the regions of the glass that are unaffected by the PIIID treatment. Since, in these regions, the polymers are only physically bound, they can leach out to the surrounding solution [72], thereby imparting antibacterial activity to it [73]. This phenomenon is expected to be more prominent in the case of the PIIID-HA discs owing to the significant thickness and porous nature of the HA discs [73,74]. Furthermore, the PIIID-generated layer on the glass surface was observed to be mildly unstable (data not reported). Surface erosion was observed from the PIIID-glass surface when they were incubated in a liquid media for a prolonged period, even within a day (data not reported). This resulted in the detachment of tiny amounts of the plasma deposited layer from the surface and their dissolution in the surrounding solution. It is conceivable that some of the attached polymers were lost to the surrounding media due to this phenomenon and that could further explain the observed antibacterial activity of the uncoated PIIID-glass surface [75].

A more significant effect was observed when the number of bacteria attached to the surface of the polymer-modified PIIID-glass surfaces was quantified (Figure 7a). A mild reduction in the number of bacteria was observed upon PIIID treatment of the surface. However, upon further polymer attachment, a 2-log_10_ (500 µg/mL polymer solution) and a 3-log_10_ (1000 µg/mL polymer solution) reduction in the number of attached bacteria was observed (Figure 7a). The density and morphology of the attached bacteria was further observed under SEM. In accordance with the quantitative assay, a higher number of bacteria was observed per unit area of the PIIID-glass surface compared to polymer-coated PIIID-glass surfaces (Figure 7c–e).

**Figure 8:**
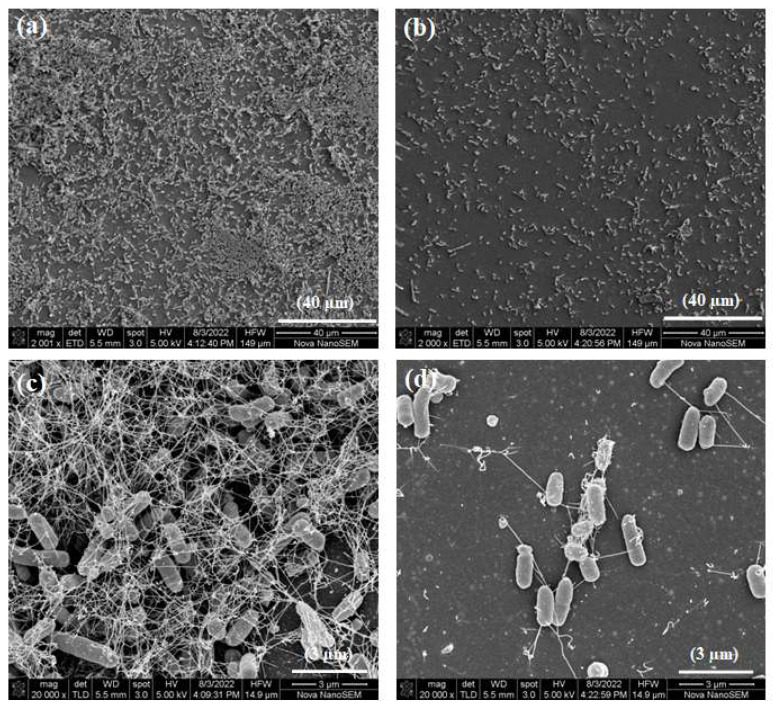
Diminished and magnified images of *E. coli* growing on PIIID-glass surfaces: (**a**,**c**) PIIID-glass coverslip, and (**b**,**d**) PIIID-glass coverslip 1000p.

On the control PIIID-glass surface, bacteria had even formed clusters that resembled the early stages of a biofilm (Figure 8a,c). No such aggregates were observed on the polymer-coated surfaces, and the few bacteria that attached were mostly found in planktonic state (Figure 8b,d). Upon closer inspection at a higher magnification, no significant surface blebbing was observed on the polymer-coated surfaces, unlike what was observed for the physically coated HA discs.

**Figure 9:**
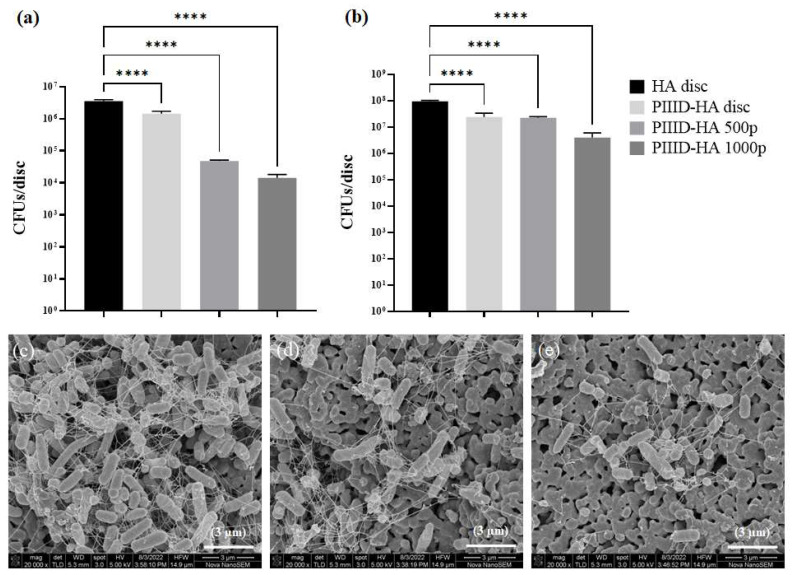
Antibacterial activity of glass coverslips covalently coated with the polymer. (**a**) Antibacterial activity on coated disc surface and (**b**) activity in the surrounding media. Scanning electron micrographs of *E. coli* growing on (**c**) PIIID-HA disc, (**d**) PIIID-HA 500p, and (**e**) PIIID-HA 1000p. **** = *p* < 0.0001.

Finally, the antibacterial activities of PIIID-treated and polymer-coated HA discs were similarly assessed. PIIID modification of the HA discs imparted some antibacterial activity to them, leading to a 0.5-log_10_ reduction in the number of viable bacteria growing in the supernatant (Figure 9b). This phenomenon was also observed on PIIID-glass coverslips, and was attributed to the desorption of the plasma coating itself. The PIIID-generated coating on HA discs was notably more stable (data not reported). Nonetheless, there could still be some desorption happening from the surface and causing mild antibacterial activity in the surrounding media [72]. Incubating PIIID-HA discs in 500 µg/mL polymer solution did not lead to an increased antibacterial activity in the supernatant. However, a further one-log_10_ reduction in the number of viable bacteria in the supernatant was observed with PIIID-HA discs that were incubated in 1000 µg/mL polymer solution (Figure 9b). This could be due to the presence of polymers inside the pores of the HA disc that did not covalently bind to the HA and diffused out into the surrounding media [73]. The reduced activity in the supernatant demonstrated by covalently modified HA surfaces compared to the physically modified surfaces could be attributed to the inability of the covalently bound polymers to leave the HA surface and diffuse into the surrounding media [76].

The antibacterial activity was found to be more prominent on the surface of the polymer-coated PIIID-HA discs (Figure 9a). Similar to the supernatant, a 0.5-log_10_ reduction in the number of viable bacteria was observed on the surface of PIIID-HA discs. This number increased to 2-log_10_ and 2.5-log_10_ in the case of PIIID-HA discs incubated with 500 µg/mL and 1000 µg/mL polymer, respectively. A dose-dependent increase in antibacterial activity was observed on the disc surfaces (Figure 9a). It can be inferred that, like the polymer-coated glass surfaces, covalent attachment of the polymer to PIIID-HA discs does not inhibit the nascent antibacterial activity of the polymer. It is also a possibility that covalently coating PIIID-HA disc and PIIID-glass surfaces with the polymer makes these surfaces bacteria repellent, and consequently, the number of bacteria that colonize the surfaces are much smaller compared to the corresponding control surfaces [77,78]. The data from the contact angle measurements performed on PIIID-glass surfaces coated with the polymer certainly hint towards changes in the surface energy of the materials upon covalent attachment of the polymer [79,80]. Further experiments are needed to segregate the contributions of the bacteria-repellent and the bactericidal/bacteriostatic or both properties of the modified surfaces in reducing the CFU counts observed in this study.

The number and morphology of the bacteria growing on the uncoated and polymer-coated PIIID-HA discs were further elucidated via SEM (Figure 9c–e). Uncoated PIIID-HA discs displayed a larger number of bacteria per unit area (Figure 9c). Furthermore, aggregations of bacteria into biofilm-like structures were observed on the same samples. The surface was colonized by both planktonic and the bacteria in these aggregates. The number of bacteria observed per unit area of the polymer-coated PIIID-HA discs was far lower than on the control PIIID-HA disc. Small groups of bacteria were observed on the disc coated with 500 μg/mL polymer (Figure 9d), but they did not resemble the biofilm-like structures observed on the control disc surface. The reduction in the number of bacteria per unit area was more pronounced on the discs coated with 1000 μg/mL polymer. Bacteria were found predominantly in their planktonic state (Figure 9e).

### 3.4. Retention of Antibacterial Activity

#### 3.4.1. XPS Analysis of Surface Elemental Composition before and after Release of Polymer

The surfaces of HA 1000p and PIIID-HA 1000p discs were analyzed via XPS after incubation in PBS for 7 days. Significant changes were observed on the surface of HA 1000p discs before and after incubation in PBS. Most notably, the nitrogen content went from 3.4% on the freshly coated disc to 0.3% on the disc incubated in PBS (Table 4), indicating a drastic reduction in the amount of the adsorbed polymer. This was further confirmed by a decrease in carbon and an increase in oxygen content on the surface (Table 4). The loss of polymer from the disc surface was correlated with the loss of activity observed in the supernatant and on the surface of HA 1000p discs after 7 days of incubation in PBS. Although changes were also observed in the elemental composition of PIIID-HA 1000p disc surface after incubation (Table 4), these changes were not as pronounced as the ones observed on HA 1000p. Therefore, the surface composition of the PIIID-HA 1000p discs did not change significantly during the incubation period. However, it is possible that polymers adsorbed inside the pores of the ceramic disc diffused out into the surface and subsequently into the surrounding media during this period. This can account for the reduction in activity observed in the supernatant after the PIIID-HA 1000p discs were incubated in PBS for 7 days. Furthermore, some of the polymer attached to the disc surface via covalent linkages could have diffused out into the surrounding media too after lysis of their bonds.

**Figure 10:**
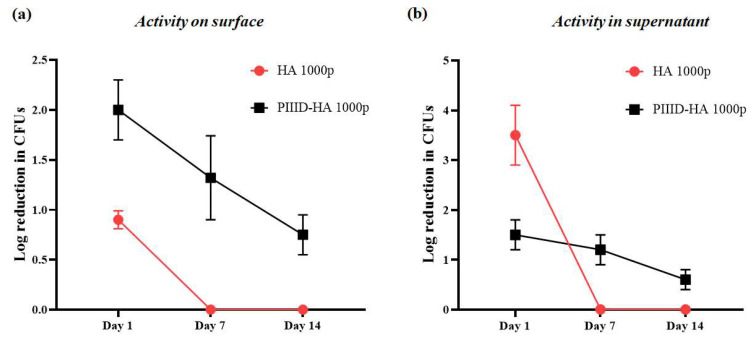
Antibacterial activity on (**a**) the surface of discs and (**b**) in the supernatant after 7 and 14 days of incubation in PBS.

The reduction in the antibacterial activity of the polymer-coated discs (both physically and covalently coated ones) was monitored over a period of 14 days. HA discs physically coated with 1000 μg/mL polymer demonstrated no antibacterial activity both in the surrounding solution and on the disc surface after 7 and 14 days of incubation in PBS. The activities of freshly coated discs measured previously were assigned to day 1. In the supernatant, the activity reduced from a 3.5-log_10_ reduction in CFUs at day 1 to a 0-log_10_ reduction in CFUs after day 7 (Figure 10a). On the disc surface, the activity reduced from an almost 1-log_10_ reduction in CFUs on day 1 to a 0-log_10_ reduction in CFUs after day 7 (Figure 10b). The lack of retention of activity can be attributed to the weak interactions between the polymers and the HA surface. These interactions are incapable of retaining the polymers on the surface of the discs for a prolonged period.

The PIIID-HA discs covalently coated with 1000 μg/mL polymer exhibited some retention of their antibacterial activity. Freshly coated discs led to a 1.5-log_10_ reduction in CFUs at day 1 in the surrounding solution. After day 7 and day 14 of incubation in PVS, the discs exhibited 1.2- and 0.6-log_10_ reductions in CFUs in the surrounding solution, respectively (Figure 10a). Similarly, activity was retained on the disc surfaces as well. After day 1, the discs exhibited a 2-log_10_ reduction in the number of CFUs on surface. This number decreased to 1.3-log_10_ and 0.7-log_10_ reductions in CFUs on the surface after day 7 and day 14, respectively (Figure 10b). Nonetheless, there was still antibacterial activity on the surface of covalently coated discs even after 14 days of incubation in PBS. The retention in activity can be attributed to the strength of covalent binding which makes it difficult for the polymers to leave the surface of the discs.

#### 3.4.2. In Vitro Inhibition of Biofilm Formation Assay

**Figure 11:**
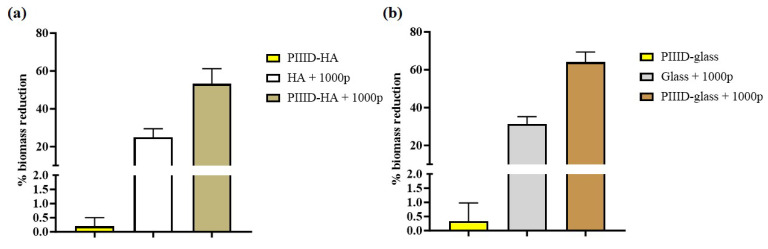
Antibiofilm activity of AP-coated HA and glass surfaces. (**a**) % biomass reduction on HA and PIIID-HA discs coated with the AP (**b**) % biomass reduction on glass and PIIID-glass coverslips coated with the AP. % biomass reduction is normalized to either blank HA disc or Glass (i.e., 0% reduction), for (**a**) and (**b**) respectively.

The ability of the polymer-coated sheets to inhibit the growth of biofilms on their surfaces was tested via crystal violet staining. Inhibition of biofilm formation was observed on the HA 1000p and PIIID-HA 1000p sheets, with the former exhibiting 24.9% and the latter exhibiting 53.16% reductions in biofilm biomass, on average, when compared to the control HA disc (Figure 11a). Inhibition of biofilm formation was also observed on coated-glass coverslips, with glass 1000p and PIIID- glass 1000p exhibiting 31.2% and 64% reductions in biofilm biomass, on average, when compared to control glass coverslip (Figure 11b). Freshly coated surfaces were tested in this study, and the anti-biofilm activity observed in this study can be attributed to the antibacterial activity of the AP present on the surface of the discs. We found a reduction in the number of CFUs on the surfaces of the polymer-coated HA and PIIID-HA discs, and also observed perturbations in bacterial morphology under SEM. These properties of the coated surfaces inhibit the formation of biofilms. The lower anti-biofilm activity observed on the surface of HA 1000p compared to that on PIIID-HA 1000p can be attributed to the gradual release of the AP from the physically coated surface over the two days of incubation in media, during which opportunistic bacteria gain access to the disc surface. A higher amount of AP is retained on the covalently coated disc surface which offers a higher degree of protection from biofilm formation.

#### 3.4.3. SEM of Biofilm Bacteria

**Figure 12:**
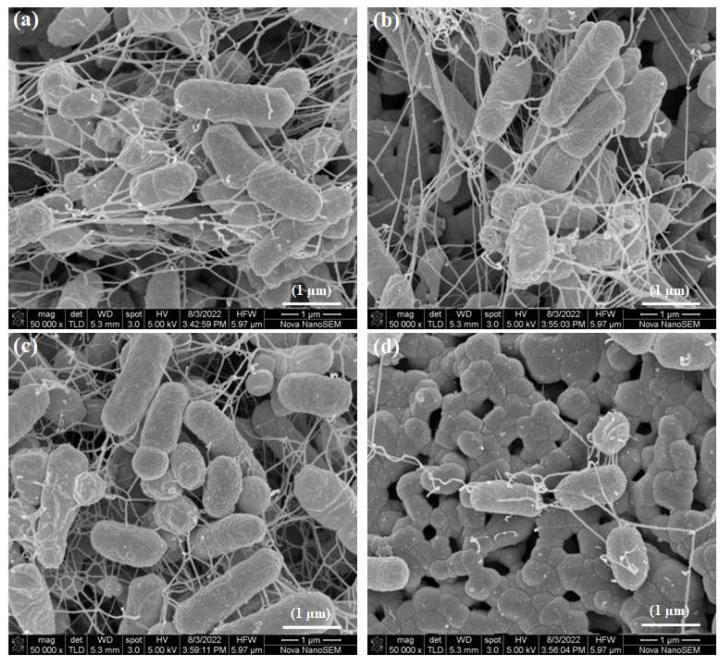
SEM of biofilm bacteria on (**a**) HA disc, (**b**) HA 1000p, (**c**) PIIID-HA, and (**d**) PIIID-HA 1000p.

The bacteria growing in biofilms on the surface of modified HA discs and glass coverslips were imaged under SEM. On HA discs, the bacteria appeared to be clustered together and with extracellular structures such as flagella extending from their bodies (Figure 12a). The bacteria growing on HA 1000p disc appeared similar in morphology to the bacteria on the control HA disc (Figure 12b).

On the PIIID-HA disc, the bacteria exhibited extracellular projections and grew in clusters (Figure 12c). On PIIID-HA 1000p, fewer bacteria were observed compared to the PIIID-HA and control HA surfaces (Figure 12d). The SEM images of biofilm bacteria on HA discs corroborate the results from the crystal violet assay.

**Figure 13:**
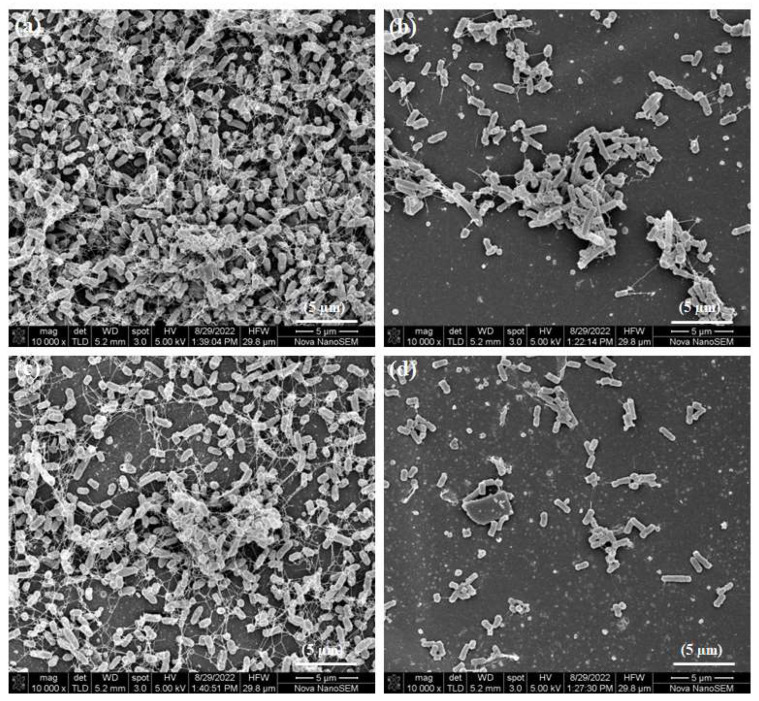
SEM of biofilm bacteria on (**a**) glass coverslip, (**b**) glass 1000p, (**c**) PIIID-glass, and (**d**) PIIID-glass 1000p.

Bacteria were found growing in clusters on glass (Figure 13a) and PIIID-glass (Figure 13c). Like the bacteria on the modified HA surfaces, the bacteria on these surfaces exhibited normal morphology and extracellular structures such as flagella. A reduction in the number of bacteria was observed on the surface of glass 1000p (Figure 13b). However, despite the reduction in number, bacterial aggregates of significant sizes were still present on the surface. A reduction in number of bacteria was accompanied by a reduction in the size of bacterial aggregates on the surface of PIIID-glass 1000p (Figure 13d). Disruption in membrane morphology of the bacteria wasn’t apparent on these surfaces. These results are in agreement with the quantitative measurements of the biofilms obtained via the crystal violet assay.

### 3.5. Cytotoxicity Assay

**Figure 14:**
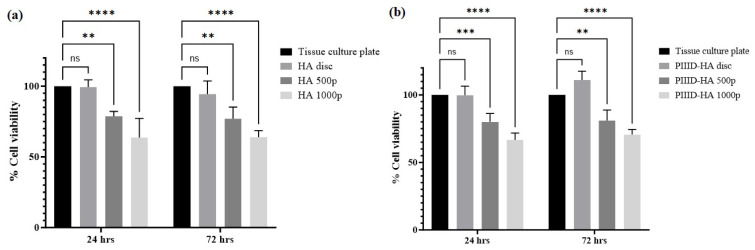
In vitro cytotoxicity of (**a**) physically coated HA discs after 24 and 72 h of cell culture, and (**b**) covalently coated PIIID-HA discs after 24 and 72 h of cell culture. ns = no significance; ** = *p* < 0.01; *** = *p* < 0.001; **** = *p* < 0.0001.

The cytotoxicity of the polymer-coated discs was measured via the alamar blue assay. Uncoated HA discs were found to be non-toxic to human fetal osteoblast cells. Upon surface coating of HA with the polymer, a dose-dependent increase in cytotoxicity was observed after 24 h of cell growth, with 79% and 64% of cells surviving, on average, on the surfaces of HA discs coated with 500 µg/mL and 1000 µg/mL polymer, respectively (Figure 14a). Similar results were found after 72 h of cell culture, too: 77% and 64% of cells survived on the surfaces of HA discs coated with 500 µg/mL and 1000 µg/mL polymer, respectively. Uncoated HA discs allowed for the survival of 94% of cells, on average (Figure 14a).

Similar trends in cytotoxicity were observed on HA discs that were covalently linked to the polymer. PIIID modification of the discs did not confer any cytotoxicity to them. Interestingly, after 72 h of culture, a higher viability of cells was observed on PIIID-HA discs (111%) than the corresponding tissue culture plate control, while after 24 h of cell culture, the viability of cells on PIIID-HA discs was nearly 100% (Figure 14b). Upon attachment of the polymer, a dose-dependent decrease in cell viability or an increase in cytotoxicity was observed. After 24 h of cell culture, cell viability was found to be 80% and 67% on PIIID-HA discs coated with 500 µg/mL and 1000 µg/mL polymer, respectively (Figure 14b). The cell viability values after 72 h of cell culture on PIIIF-HA discs coated with 500 µg/mL and 1000 µg/mL polymer were found to be 81% and 71%, respectively (Figure 14b).

## 4. Discussion

The prevalence of ODRIs demands concerted effort by researchers [1,3]. The loss of effectiveness of traditional antibiotics owing to the rapid evolution of antibiotic-resistant bacteria and the concomitant dearth of novel antibiotics being developed has shifted the focus of researchers towards alternative antimicrobial strategies [10,11,30]. AMPs and their mimics have garnered a lot of attention as potential alternatives to traditional antibiotics [14,30]. Of these, mimics of AMPs appear to be more promising therapeutic agents due to their favorable pharmacological properties alongside their antibacterial activity [28,30]. Polymeric mimics of AMPs, also known as APs, offer exciting opportunities for the development of novel strategies to combat ODRIs [32]. In this study, we characterized the antibacterial properties of an AP-coated HA discs and glass coverslips against the Gram-negative pathogen *E. coli*. The AP used in the current study has previously demonstrated significant antibacterial activity against the Gram-negative pathogen in solution [39,40,41]. However, to the best of our knowledge, ours is the first study to explore the effectiveness of the AP as an antibacterial coating for materials of significance in orthopedics research.

The AP was successfully coated on the surface of HA discs via physical and covalent attachments and on glass coverslips via covalent attachment. Physical attachment of antibacterial compounds to biomaterials has been a common strategy for imparting antibacterial properties to biomaterials [81,82]. Physical attachment is usually accomplished via adsorption or entrapment of the antibacterial compounds [82]. Adsorption is facilitated by the charge distributions within the antibacterial entity and the biomaterial surface. The AP used in this study is cationic at physiological pH, whereas the biomaterial, HA, is predominantly negatively charged. Physical attachment was thus achieved via electrostatic interactions between the AP and the HA disc surface. Covalent attachment is another commonly employed strategy for the conjugation of antibacterial compounds to biomaterials [82]. The traditional methods of covalent conjugation usually involve multiple reaction steps that often leave behind toxic remnants [82,83]. PIIID has emerged as an exciting technology for the attachment of desirable compounds to the surfaces of biomaterials [63]. Unlike traditional linker-based approaches, PIIID is a single-step process and is relatively easy to perform. In a recent study, Tran et al. demonstrated the efficacy of PIIID treatment in covalently attaching an AMP to the surface of biomaterials [61]. In this study, we utilized the PIIID technique for covalent attachment of the AP to HA and glass. The two modes of attachment (physical and covalent) often yield materials with varying antibacterial properties owing to differences in the orientation, diffusion, release, and other physicochemical characteristics of antibacterial compounds from coated materials [84]. Both types of attachment were first confirmed via XPS. XPS is a reliable technique for determining the chemical composition on a materials surface and a previous study has demonstrated the efficacy of XPS in confirming the attachment of AMPs to PIIID-treated surfaces [61]. Attachment to PIIID-glass was further confirmed via contact angle measurements.

Physically coated HA discs exhibited significantly higher antibacterial activity in the supernatant compared to the surface of the discs (which also showed a reduction in CFUs). This can be attributed to the leaching out of the AP from the discs [85]. Physically conjugated antibacterial biomaterials have been used for local delivery of antibacterial entities [85]. Diffusion is one of the key phenomena exploited by these systems for the sustained delivery of antibacterial compounds [85], the other being degradation of the carrier material. Since the HA discs used in this study were not degradable under physiological conditions, it is reasonable to attribute the leaching out of the AP to diffusion of the AP from the pores and from the surface of the discs to the surrounding media. Nonetheless, not all of the AP leached out into the surrounding media during the antibacterial assay, as activity was also observed on the surface of the discs. The morphology of *E. coli* attached to the surface of AP-coated HA discs appeared to be distorted, while those on the control HA disc appeared normal. This indicates that the AP, in its physically bound state, could be killing bacteria by disrupting their membranes [86,87]. Membrane disruption has been reported as one of the modes of antibacterial activity exhibited by AMP-mimicking APs [88]; however, further investigation of the mode of action of the AP used in this study (in its surface-bound state) needs to be performed to substantiate any hypothesis about its mechanism of antibacterial activity.

Covalently coated PIIID-HA discs and glass coverslips exhibited significantly higher antibacterial activity on their surfaces compared to the supernatants. This can be attributed to the retention of the AP on the covalently coated surfaces. Further characterization via XPS confirmed that the covalently coated PIIID-HA discs successfully retained the surface-bound AP even after 7 days of incubation in PBS, which was not the case for the physically coated HA discs. Interestingly, no disruption in membrane morphology was observed on the bacteria attached to the covalently coated surfaces. This could be attributed to different modes of action exhibited by the AP in their physically bound and covalently bound states but will need to be tested experimentally via membrane disruption, depolarization assays.

The anti-biofilm activity of the physically and covalently coated discs and glass surfaces was quantified. AP coating (both physical and covalent) resulted in a significant reduction in the magnitude of biofilm formation. However, the degree of reduction in biofilm formation was much higher on the covalently coated surfaces compared to the physically coated ones. This can once again be attributed to the higher retention of the AP on the covalently coated surfaces. SEM images of the biofilms on the control surfaces revealed large clumps of bacteria, while only single bacteria and small clumps of bacteria were visible on the AP-coated surfaces. Further characterizations of the mechanism of action of the AP in physically bound and covalently bound states should reveal some interesting results. Nonetheless, the AP investigated in this study is a promising candidate for controlling *E. coli*-mediated infections on orthopedic biomaterial surfaces.

## 5. Conclusions

In summary, we successfully synthesized antibacterial coatings for HA surfaces via two different techniques: physical and covalent attachment of APs. The detailed mechanism of action of these coatings needs to be understood further, but the coatings demonstrated significant antibacterial activity against the Gram-negative pathogen *E. coli.* Although both forms of coating yielded surfaces with antibacterial activity, covalently coated surfaces retained their activity over a longer period of time compared to physically coated surfaces. This work paves the way for further research into PIIID modification of orthopedic surfaces for the attachment of biologically relevant molecules, including novel antimicrobial entities.

## Figures and Tables

**Table 1:** Elemental composition of various HA surfaces determined via XPS.

Elemental Composition (% of Total)	HA	HA 500p	HA 1000p	PIIID-HA	PIIID-HA 500p	PIIID-HA 1000p
Carbon	18.2	28.9	26.9	66.4	65.5	66.2
Oxygen	51.1	43.1	44.6	9.3	14.8	14.3
Nitrogen	0.1	3.7	3.4	23.1	18.2	17.8
Calcium	17.8	13.2	13.7	0.6	0.5	0.7
Phosphorus	10	7.9	8.2	0.4	0.3	0.5

**Table 2:** Elemental composition of various glass surfaces determined via XPS.

Elemental Composition (% of Total)	Glass Coverslip	PIIID-Glass Coverslip	PIIID-Glass Coverslip 500p	PIIID-Glass Coverslip 1000p
Carbon	21.2	73.6	58.7	59.7
Oxygen	55.4	12.3	19.7	20
Nitrogen	0	12.3	7.1	6.1
Silicon	14.4	0	0	0

**Table 3:** Elemental composition of various PIIID-HA surfaces determined via XPS before and after SDS treatment.

Elemental Composition (% of Total)	PIIID-HA	PIIID-HA 500p	PIIID-HA 1000p	PIIID-HA + SDS	PIIID-HA 500p + SDS	PIIID-HA 1000p + SDS
Carbon	66.4	65.5	66.2	66.6	65.2	66.4
Oxygen	9.3	15	14.3	9.3	14.4	14.5
Nitrogen	23.1	18.8	17.8	23.2	18.4	17.8
Calcium	0.6	0.5	0.7	0.7	0.7	0.7
Phosphorus	0.4	0.3	0.5	0.3	0.6	0.7

**Table 4:** Elemental composition of AP-coated samples before and after incubation in PBS.

Elemental Composition (% of Total)	HA + 1000p		PIIID-HA + 1000p	
	Before Incubation	After Incubation	Before Incubation	After Incubation
Carbon	26.9	19.1	66.2	68
Oxygen	44.6	52.8	14.3	13.1
Nitrogen	3.4	0.3	17.8	16.5
Calcium	13.2	15.6	0.7	0.5
Phosphorus	7.9	12.6	0.5	0.8

## Data Availability

The data presented in the study is available in the article and Supplementary Information is available online.

## Supplementary Materials

The following supporting information can be downloaded at: https://www.mdpi.com/article/10.3390/ma16145045/s1, Figure S1: (a) FT-IR spectra of HA disc, PIIID-HA disc, PIIID-HA 500p, and PIIID-HA 1000p spanning the entire range of detection (45–4000 cm^−1^), (b) RAMAN spectra of HA disc, PIIID-HA disc, PIIID-HA 500p, and PIIID-HA 1000p spanning the entire range of detection (50–1800 cm^−1^), (c) RAMAN spectra of pure HA disc spanning the entire range of detection (50–1800 cm^−1^); Figure S2: (a) XPS spectrum and peak-differentiation-imitating analysis of (b) C1s, (c) O1s, and (d) N1s species of HA discs; Figure S3: (a) XPS spectrum and peak-differentiation-imitating analysis of (b) C1s, (c) O1s, and (d) N1s species of HA 500p; Figure S4: (a) XPS spectrum and peak-differentiation-imitating analysis of (b) C1s, (c) O1s, and (d) N1s species of HA 1000p; Figure S5: (a) XPS spectrum and peak-differentiation-imitating analysis of (b) C1s, (c) O1s, and (d) N1s species of PIIID-HA discs; Figure S6: (a) XPS spectrum and peak-differentiation-imitating analysis of (b) C1s, (c) O1s, and (d) N1s species of PIIID-HA 500p; Figure S7: (a) XPS spectrum and peak-differentiation-imitating analysis of (b) C1s, (c) O1s, and (d) N1s species of PIIID-HA 1000p.

## References

[1] Moriarty T.F., Kuehl R., Coenye T., Metsemakers W.-J., Morgenstern M., Schwarz E.M., Riool M., Zaat S.A., Khana N., Kates S.L. (2017). Orthopaedic device-related infection: Current and future interventions for improved prevention and treatment. EFORT Open Rev..

[2] Saadatian-Elahi M., Teyssou R., Vanhems P. (2008). *Staphylococcus aureus*, the major pathogen in orthopaedic and cardiac surgical site infections: A literature review. Int. J. Surg..

[3] Crémet L., Corvec S., Bémer P., Bret L., Lebrun C., Lesimple B., Miegeville A.-F., Reynaud A., Lepelletier D., Caroff N. (2012). Orthopaedic-implant infections by *Escherichia coli*: Molecular and phenotypic analysis of the causative strains. J. Infect..

[4] Crémet L., Broquet A., Jacqueline C., Chaillou C., Asehnoune K., Corvec S., Caroff N. (2016). Innate immune evasion of *Escherichia coli* clinical strains from orthopedic implant infections. Eur. J. Clin. Microbiol. Infect. Dis..

[5] Crémet L., Broquet A., Brulin B., Jacqueline C., Dauvergne S., Brion R., Asehnoune K., Corvec S., Heymann D., Caroff N. (2015). Pathogenic potential of *Escherichia coli* clinical strains from orthopedic implant infections towards human osteoblastic cells. Pathog. Dis..

[6] Zimmerli W. (2014). Clinical presentation and treatment of orthopaedic implant-associated infection. J. Intern. Med..

[7] Spitzmüller R., Gümbel D., Güthoff C., Zaatreh S., Klinder A., Napp M., Bader R., Mittelmeier W., Ekkernkamp A., Kramer A. (2019). Duration of antibiotic treatment and risk of recurrence after surgical management of orthopaedic device infections: A multicenter case-control study. BMC Musculoskelet. Disord..

[8] Darouiche R.O. (2004). Treatment of infections associated with surgical implants. N. Engl. J. Med..

[9] van de Belt H., Neut D., Schenk W., van Horn J.R., van der Mei H.C., Busscher H.J. (2001). Infection of orthopedic implants and the use of antibiotic-loaded bone cements: A review. Acta Orthop. Scand..

[10] (2013). The antibiotic alarm. Nature.

[11] Ventola C.L. (2015). The antibiotic resistance crisis: Part 1: Causes and threats. Pharm. Ther..

[12] Browne K., Chakraborty S., Chen R., Willcox M.D., Black D.S., Walsh W.R., Kumar N. (2020). A new era of antibiotics: The clinical potential of antimicrobial peptides. Int. J. Mol. Sci..

[13] Zhang J., Liu X., Li H., Chen C., Bin Hu B., Niu X., Li Q., Zhao B., Xie Z., Wang Y. (2016). Exosomes/tricalcium phosphate combination scaffolds can enhance bone regeneration by activating the pi3k/akt signaling pathway. Stem Cell Res. Ther..

[14] Magana M., Pushpanathan M., Santos A.L., Leanse L., Fernandez M., Ioannidis A., Giulianotti M.A., Apidianakis Y., Bradfute S., Ferguson A.L. (2020). The value of antimicrobial peptides in the age of resistance. Lancet Infect. Dis..

[15] Seyfi R., Kahaki F.A., Ebrahimi T., Montazersaheb S., Eyvazi S., Babaeipour V., Tarhriz V. (2020). Antimicrobial peptides (amps): Roles, functions and mechanism of action. Int. J. Pept. Res. Ther..

[16] Pushpanathan M., Gunasekaran P., Rajendhran J. (2013). Antimicrobial peptides: Versatile biological properties. Int. J. Pept..

[17] Pasupuleti M., Schmidtchen A., Malmsten M. (2012). Antimicrobial peptides: Key components of the innate immune system. Crit. Rev. Biotechnol..

[18] Diamond G., Beckloff N., Weinberg A., Kisich K.O. (2009). The roles of antimicrobial peptides in innate host defense. Curr. Pharm. Des..

[19] Bechinger B., Gorr S.-U. (2016). Antimicrobial peptides: Mechanisms of action and resistance. J. Dent. Res..

[20] Kumar P., Kizhakkedathu J.N., Straus S.K. (2018). Antimicrobial peptides: Diversity, mechanism of action and strategies to improve the activity and biocompatibility in vivo. Biomolecules.

[21] Wenzel M., Chiriac A.I., Otto A., Zweytick D., May C., Schumacher C., Gust R., Albada H.B., Penkova M., Kraemer U. (2014). Small cationic antimicrobial peptides delocalize peripheral membrane proteins. Proc. Natl. Acad. Sci. USA.

[22] Hollmann A., Martinez M., Maturana P., Semorile L.C., Maffia P.C. (2018). Antimicrobial peptides: Interaction with model and biological membranes and synergism with chemical antibiotics. Front. Chem..

[23] Chen C.H., Lu T.K. (2020). Development and challenges of antimicrobial peptides for therapeutic applications. Antibiotics.

[24] Zeng Z.-Z., Huang S.-H., Alezra V., Wan Y. (2021). Antimicrobial peptides: Triumphs and challenges. Futur. Med. Chem..

[25] Jiang Y., Chen Y., Song Z., Tan Z., Cheng J. (2021). Recent advances in design of antimicrobial peptides and polypeptides toward clinical translation. Adv. Drug Deliv. Rev..

[26] Molchanova N., Hansen P.R., Franzyk H. (2017). Advances in development of antimicrobial peptidomimetics as potential drugs. Molecules.

[27] Watson E., Tatara A.M., Kontoyiannis D.P., Mikos A.G. (2017). Inherently antimicrobial biodegradable polymers in tissue engineering. ACS Biomater. Sci. Eng..

[28] Thaker H.D., Som A., Ayaz F., Lui D., Pan W., Scott R.W., Anguita J., Tew G.N. (2012). Synthetic mimics of antimicrobial peptides with immunomodulatory responses. J. Am. Chem. Soc..

[29] Mendez-Samperio P. (2014). Peptidomimetics as a new generation of antimicrobial agents: Current progress. Infect. Drug Resist..

[30] Rotem S., Mor A. (2009). Antimicrobial peptide mimics for improved therapeutic properties. Biochim. Biophys. Acta.

[31] Sztukowska M.N., Roky M., DeMuth D.R. (2019). Peptide and non-peptide mimetics as potential therapeutics targeting oral bacteria and oral biofilms. Mol. Oral Microbiol..

[32] Kuroda K., Caputo G.A. (2013). Antimicrobial polymers as synthetic mimics of host-defense peptides. Wiley Interdiscip. Rev. Nanomed. Nanobiotechnol..

[33] Carmona-Ribeiro A.M., Araújo P.M. (2021). Antimicrobial polymer-based assemblies: A review. Int. J. Mol. Sci..

[34] Huang K.-S., Yang C.-H., Huang S.-L., Chen C.-Y., Lu Y.-Y., Francolini I. (2016). Recent advances in antimicrobial polymers: A mini-review. Int. J. Mol. Sci..

[35] Lam S.J., O’Brien-Simpson N.M., Pantarat N., Sulistio A., Wong E.H.H., Chen Y.-Y., Lenzo J.C., Holden J.A., Blencowe A., Reynolds E.C. (2016). Combating multidrug-resistant gram-negative bacteria with structurally nanoengineered antimicrobial peptide polymers. Nat. Microbiol..

[36] Wong E.H.H., Khin M.M., Ravikumar V., Si Z., Rice S.A., Chan-Park M. (2016). Modulating antimicrobial activity and mammalian cell biocompatibility with glucosamine-functionalized star polymers. Biomacromolecules.

[37] Yang C., Krishnamurthy S., Liu J., Liu S., Lu X., Coady D.J., Cheng W., De Libero G., Singhal A., Hedrick J.L. (2016). Broad-spectrum antimicrobial star polycarbonates functionalized with mannose for targeting bacteria residing inside immune cells. Adv. Health Mater..

[38] Yuan W., Wei J., Lu H., Fan L., Du J. (2012). Water-dispersible and biodegradable polymer micelles with good antibacterial efficacy. Chem. Commun..

[39] Nguyen T.-K., Lam S.J., Ho K.K.K., Kumar N., Qiao G.G., Egan S., Boyer C., Wong E.H.H. (2017). Rational design of single-chain polymeric nanoparticles that kill planktonic and biofilm bacteria. ACS Infect. Dis..

[40] Namivandi-Zangeneh R., Kwan R.J., Nguyen T.-K., Yeow J., Byrne F.L., Oehlers S.H., Wong E.H.H., Boyer C. (2018). The effects of polymer topology and chain length on the antimicrobial activity and hemocompatibility of amphiphilic ternary copolymers. Polym. Chem..

[41] Namivandi-Zangeneh R., Sadrearhami Z., Dutta D., Willcox M., Wong E.H.H., Boyer C. (2019). Synergy between synthetic antimicrobial polymer and antibiotics: A promising platform to combat multidrug-resistant bacteria. ACS Infect. Dis..

[42] Awasthi S., Pandey S.K., Arunan E., Srivastava C. (2021). A review on hydroxyapatite coatings for the biomedical applications: Experimental and theoretical perspectives. J. Mater. Chem. B.

[43] Petit R. (1999). The use of hydroxyapatite in orthopaedic surgery: A ten-year review. Eur. J. Orthop. Surg. Traumatol..

[44] Shepherd J., Friederichs R.J., Best S.M., Mucalo M. (2015). Synthetic hydroxyapatite for tissue engineering applications. Hydroxyapatite (Hap) for Biomedical Applications.

[45] Shao H., He J., Lin T., Zhang Z., Zhang Y., Liu S. (2019). 3d gel-printing of hydroxyapatite scaffold for bone tissue engineering. Ceram. Int..

[46] Mondal S., Pal U. (2019). 3d hydroxyapatite scaffold for bone regeneration and local drug delivery applications. J. Drug Deliv. Sci. Technol..

[47] Harun W.S.W., Asri R.I.M., Sulong A.B., Ghani S.A.C., Ghazalli Z., Thirumalai J. (2018). Chapter 5: Hydroxyapatite-based coating on biomedical implant. Hydroxyapatite: Advances in Composite Nanomaterials, Biomedical Applications and Its Technological Facets.

[48] Ben-Nissan B., Choi A., Roest R., Latella B., Bendavid A., Mucalo M. (2015). Adhesion of hydroxyapatite on titanium medical implants. Hydroxyapatite (Hap) for Biomedical Applications.

[49] Bal Z., Kaito T., Korkusuz F., Yoshikawa H. (2019). Bone regeneration with hydroxyapatite-based biomaterials. Emergent Mater..

[50] Kolmas J., Groszyk E., Kwiatkowska-Różycka D. (2014). Substituted hydroxyapatites with antibacterial properties. BioMed Res. Int..

[51] Iqbal F., Fatima H., Khan A.S., Chaudhry A.A. (2020). Coating of hydroxyapatite and substituted apatite on dental and orthopedic implants. Handbook of Ionic Substituted Hydroxyapatites.

[52] Kanhed S., Awasthi S., Midha S., Nair J., Nisar A., Patel A.K., Pandey A., Sharma R., Goel S., Upadhyaya A. (2018). Microporous hydroxyapatite ceramic composites as tissue engineering scaffolds: An experimental and computational study. Adv. Eng. Mater..

[53] Gutiérrez-Prieto S.J., Fonseca L.F., Sequeda-Castañeda L.G., Díaz K.J., Castañeda L.Y., Leyva-Rojas J.A., Salcedo-Reyes J.C., Acosta A.P. (2019). Elaboration and biocompatibility of an eggshell-derived hydroxyapatite material modified with si/plga for bone regeneration in dentistry. Int. J. Dent..

[54] Yamamura H., da Silva V.H.P., Ruiz P., Ussui V., Lazar D.R.R., Renno A.C.M., Ribeiro D.A. (2018). Physico-chemical characterization and biocompatibility of hydroxyapatite derived from fish waste. J. Mech. Behav. Biomed. Mater..

[55] Marinescu C., Sofronia A., Anghel E.M., Baies R., Constantin D., Seciu A.-M., Gingu O., Tanasescu S. (2019). Microstructure, stability and biocompatibility of hydroxyapatite–titania nanocomposites formed by two step sintering process. Arab. J. Chem..

[56] Sathiyavimal S., Vasantharaj S., LewisOscar F., Selvaraj R., Brindhadevi K., Pugazhendhi A. (2020). Natural organic and inorganic–hydroxyapatite biopolymer composite for biomedical applications. Prog. Org. Coat..

[57] Townsend L., Williams R.L., Anuforom O., Berwick M.R., Halstead F., Hughes E., Stamboulis A., Oppenheim B., Gough J., Grover L. (2017). Antimicrobial peptide coatings for hydroxyapatite: Electrostatic and covalent attachment of antimicrobial peptides to surfaces. J. R. Soc. Interface.

[58] Huang Z.-B., Shi X., Mao J., Gong S.-Q. (2016). Design of a hydroxyapatite-binding antimicrobial peptide with improved retention and antibacterial efficacy for oral pathogen control. Sci. Rep..

[59] Alotaibi N.H., Munir M.U., Alruwaili N.K., Alharbi K.S., Ihsan A., Almurshedi A.S., Khan I.U., Bukhari S.N.A., Rehman M., Ahmad N. (2022). Synthesis and characterization of antibiotic-loaded biodegradable citrate functionalized mesoporous hydroxyapatite nanocarriers as an alternative treatment for bone infections. Pharmaceutics.

[60] Chakraborty S., Kuppusamy R., Roohani I., Walsh W.R., Willcox M.D.P., Kumar N., Chen R. (2021). Antibacterial peptidomimetic and characterization of its efficacy as an antibacterial and biocompatible coating for bioceramic-based bone substitutes. Mater. Adv..

[61] Tran C.T.H., Yasir M., Dutta D., Eswaramoorthy N., Suchowerska N., Willcox M., McKenzie D.R. (2019). Single step plasma process for covalent binding of antimicrobial peptides on catheters to suppress bacterial adhesion. ACS Appl. Bio Mater..

[62] Bilek M.M., McKenzie D.R. (2010). Plasma modified surfaces for covalent immobilization of functional biomolecules in the absence of chemical linkers: Towards better biosensors and a new generation of medical implants. Biophys. Rev..

[63] Yin Y., Bilek M.M., McKenzie D.R., Nosworthy N.J., Kondyurin A., Youssef H., Byrom M.J., Yang W. (2009). Acetylene plasma polymerized surfaces for covalent immobilization of dense bioactive protein monolayers. Surf. Coat. Technol..

[64] Lu T., Qiao Y., Liu X. (2012). Surface modification of biomaterials using plasma immersion ion implantation and deposition. Interface Focus.

[65] Katsifis G.A., Suchowerska N., McKenzie D.R. (2020). Quantification of dose in plasma immersion ion implantation of polymer bone scaffolds: Probe diagnostics of a pulsed dielectric barrier discharge. Plasma Process Polym..

[66] Moad G., Rizzardo E., Thang S.H. (2013). Raft polymerization and some of its applications. Chem. Asian J..

[67] Boyer C., Bulmus V., Davis T.P., Ladmiral V., Liu J., Perrier S. (2009). Bioapplications of raft polymerization. Chem. Rev..

[68] Balouiri M., Sadiki M., Ibnsouda S.K. (2016). Methods for in vitro evaluating antimicrobial activity: A review. J. Pharm. Anal..

[69] Deng L., Deng Y., Xie K. (2017). Agnps-decorated 3d printed peek implant for infection control and bone repair. Colloids Surf. B Biointerfaces.

[70] Tran C.T., Raeber T.J., Murdoch B.J., Barlow A.J., Partridge J.G., McCulloch D.G., McKenzie D.R. (2019). Conducting carbon films with covalent binding sites for biomolecule attachment. J. Appl. Phys..

[71] Seah M.P. (1984). A review of the analysis of surfaces and thin films by AES and XPS. Vacuum.

[72] Zhang W., Wang X.-C., Wang J.-J., Zhang L.-L. (2019). Drugs adsorption and release behavior of collagen/bacterial cellulose porous microspheres. Int. J. Biol. Macromol..

[73] Simovic S., Losic D., Vasilev K. (2010). Controlled drug release from porous materials by plasma polymer deposition. Chem. Commun..

[74] Gao J., Huang J., Shi R., Wei J., Lei X., Dou Y., Li Y., Zuo Y., Li J. (2021). Loading and releasing behavior of selenium and doxorubicin hydrochloride in hydroxyapatite with different morphologies. ACS Omega.

[75] Zuleger S., Lippold B.C. (2001). Polymer particle erosion controlling drug release. I. Factors influencing drug release and characterization of the release mechanism. Int. J. Pharm..

[76] Püntener M., Fibbioli M., Bakker E., Pretsch E. (2002). Response and diffusion behavior of mobile and covalently immobilized H^+^-ionophores in polymeric membrane ion-selective electrodes. Electroanalysis.

[77] Shao W., Ma H., Yu T., Wu C., Hong Z., Xiong Y., Xie Q. (2020). Antifouling pvdf membrane by surface covalently anchoring functionalized graphene quantum dots. Ind. Eng. Chem. Res..

[78] Han J.-L., Xia X., Tao Y., Yun H., Hou Y.-N., Zhao C.-W., Luo Q., Cheng H.-Y., Wang A.-J. (2016). Shielding membrane surface carboxyl groups by covalent-binding graphene oxide to improve anti-fouling property and the simultaneous promotion of flux. Water Res..

[79] Del Grosso C.A., Leng C., Zhang K., Hung H.-C., Jiang S., Chen Z., Wilker J.J. (2020). Surface hydration for antifouling and bio-adhesion. Chem. Sci..

[80] Chang J., He X., Yang Z., Bai X., Wood R.J., Wharton J.A., Lu P., Yuan C. (2022). Surface topography effects on the wettability and antifouling performance of nano-zno epoxy composite coatings. Surf. Coat. Technol..

[81] Francolini I., Vuotto C., Piozzi A., Donelli G. (2017). Antifouling and antimicrobial biomaterials: An overview. Apmis.

[82] Pahlevanzadeh F., Setayeshmehr M., Bakhsheshi-Rad H.R., Emadi R., Kharaziha M., Poursamar S.A., Ismail A.F., Sharif S., Chen X., Berto F. (2022). A review on antibacterial biomaterials in biomedical applications: From materials perspective to bioinks design. Polymers.

[83] Chen C.-P., Jing R.-Y., Wickstrom E. (2017). Covalent attachment of daptomycin to ti6al4v alloy surfaces by a thioether linkage to inhibit colonization by *Staphylococcus aureus*. ACS Omega.

[84] Sadowska J.M., Genoud K.J., Kelly D.J., O’Brien F.J. (2021). Bone biomaterials for overcoming antimicrobial resistance: Advances in non-antibiotic antimicrobial approaches for regeneration of infected osseous tissue. Mater. Today.

[85] ter Boo G.-J.A., Grijpma D.W., Moriarty T.F., Richards R.G., Eglin D. (2015). Antimicrobial delivery systems for local infection prophylaxis in orthopedic- and trauma surgery. Biomaterials.

[86] Lin L., Chi J., Yan Y., Luo R., Feng X., Zheng Y., Xian D., Li X., Quan G., Liu D. (2021). Membrane-disruptive peptides/peptidomimetics-based therapeutics: Promising systems to combat bacteria and cancer in the drug-resistant era. Acta Pharm. Sin. B.

[87] Xiao F., Cao B., Wang C., Guo X., Li M., Xing D., Hu X. (2019). Pathogen-specific polymeric antimicrobials with significant membrane disruption and enhanced photodynamic damage to inhibit highly opportunistic bacteria. ACS Nano.

[88] Lin M., Sun J. (2022). Antimicrobial peptide-inspired antibacterial polymeric materials for biosafety. Biosaf. Health.

