# Supplementary material for: Electrostatic and Covalent Binding of an Antibacterial Polymer to Hydroxyapatite for Protection against Escherichia coli Colonization
Source: Materials (Basel). 2023 Jul 17;16(14):5045. doi: 10.3390/ma16145045 (PMC10385198; doi:10.3390/ma16145045)
Supplement: Supplementary file 1 [file materials-16-05045-s001.zip › materials-2485450-supplementary.pdf]

Supplementary information

Electrostatic and covalent binding of an antibacterial polymer to hydroxyapatite for protection against *Escherichia coli* colonization

Sudip Chakraborty [1], Georgio Katsifis [2], Iman Roohani [1,3,4], Cyrille Boyer [5], David McKenzie [2,3], Mark D. P. Willcox [6], Renxun Chen [1,*], Naresh Kumar [1,*],

[1] School of Chemistry, UNSW Sydney, 2052, Kensington, NSW, Australia;

[2] School of Physics, University of Sydney, Sydney, NSW 2006, Australia;

[3] Charles Perkins Centre, University of Sydney, Sydney, NSW 2006, Australia;

[4] School of Life and Environmental Sciences, University of Sydney, Sydney, NSW 2006, Australia

[5] School of Chemical Engineering, UNSW Sydney, 2052, Kensington, NSW, Australia;

[6] School of Optometry and Vision Science, UNSW Sydney, 2052, Kensington, NSW, Australia.;

* Correspondence: r.chen@unsw.edu.au (RC); n.kumar@unsw.edu.au (NK)

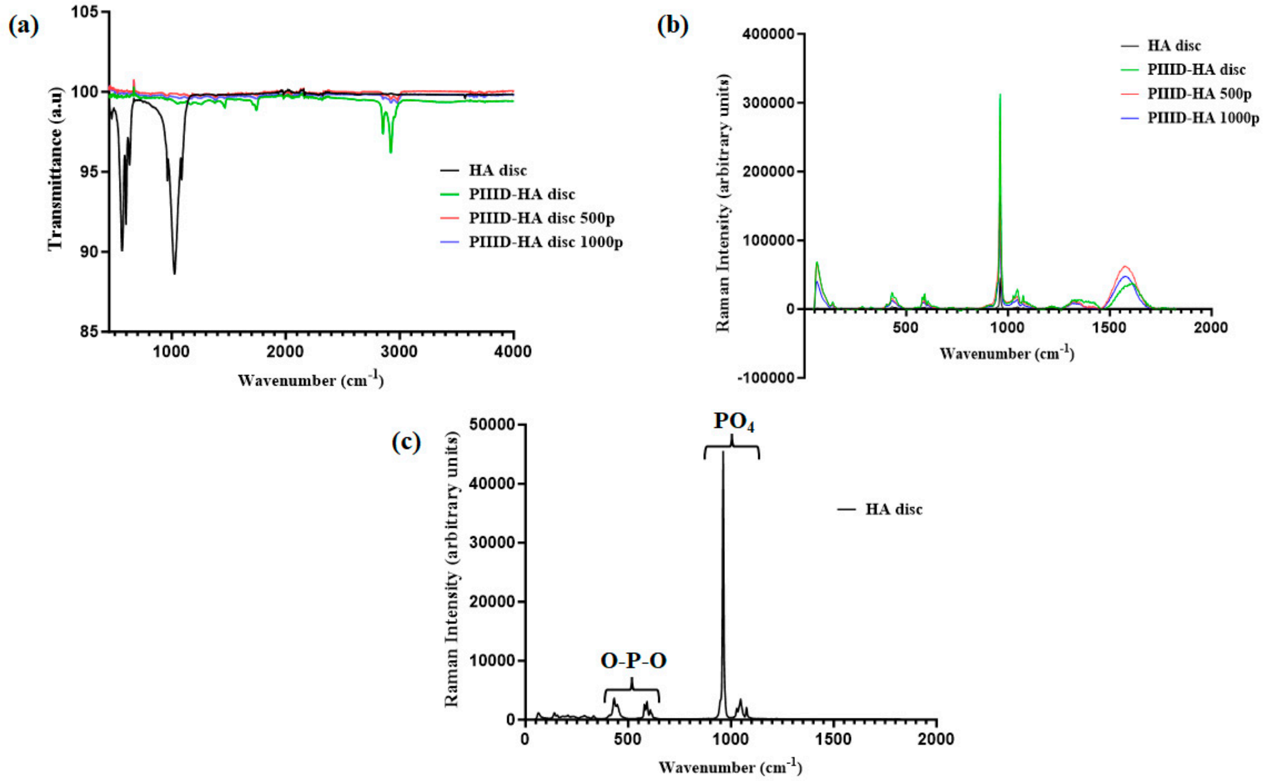

Figure S1. (a) FT-IR spectra of HA disc, PIIID-HA disc, PIIID-HA 500p, and PIIID-HA 1000p spanning the entire range of detection (45-4000 cm^{-1}), (b) RAMAN spectra of HA disc, PIIID-HA disc, PIIID-HA 500p, and PIIID-HA 1000p spanning the entire range of detection (50-1800 cm^{-1}), (c) RAMAN spectra of pure HA disc spanning the entire range of detection (50-1800 cm^{-1})

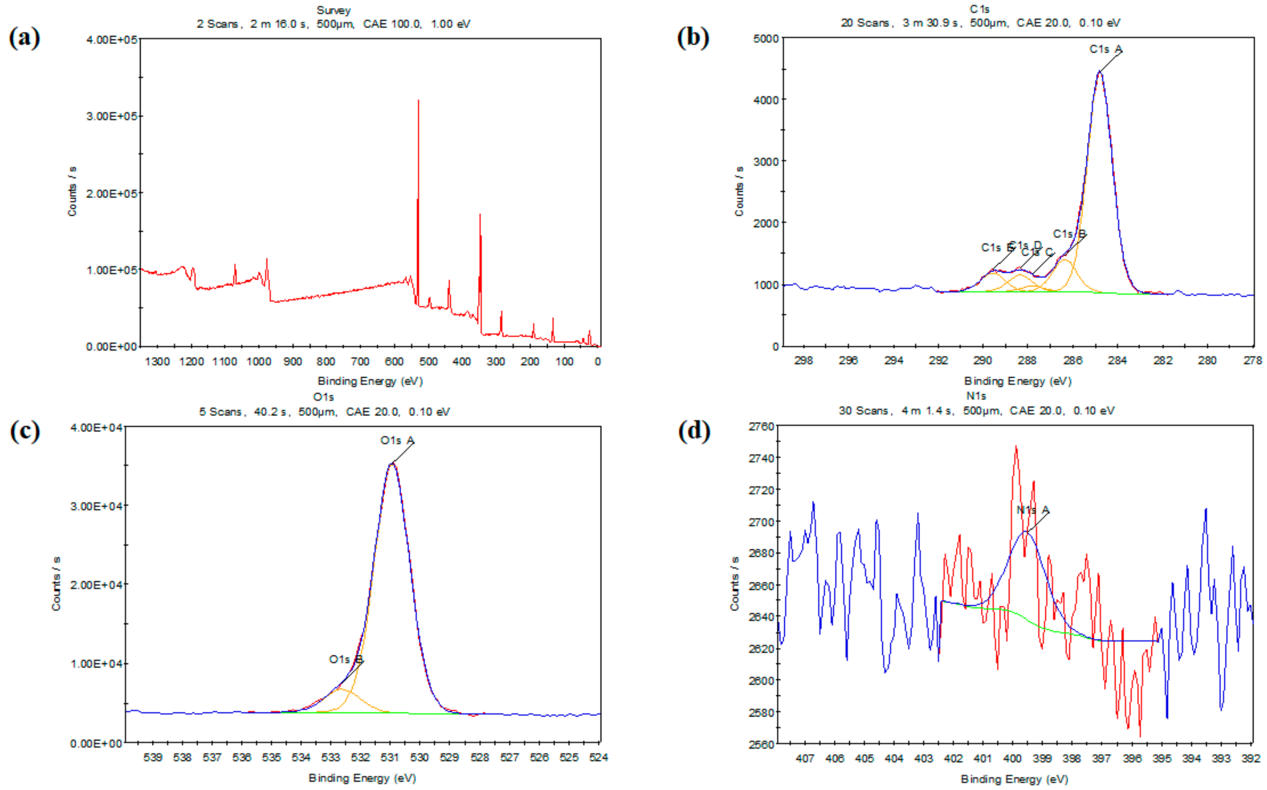

Figure S2. (a) XPS spectrum and peak-differentiation-imitating analysis of (b) C1s, (c) O1s, and (d) N1s species of HA discs

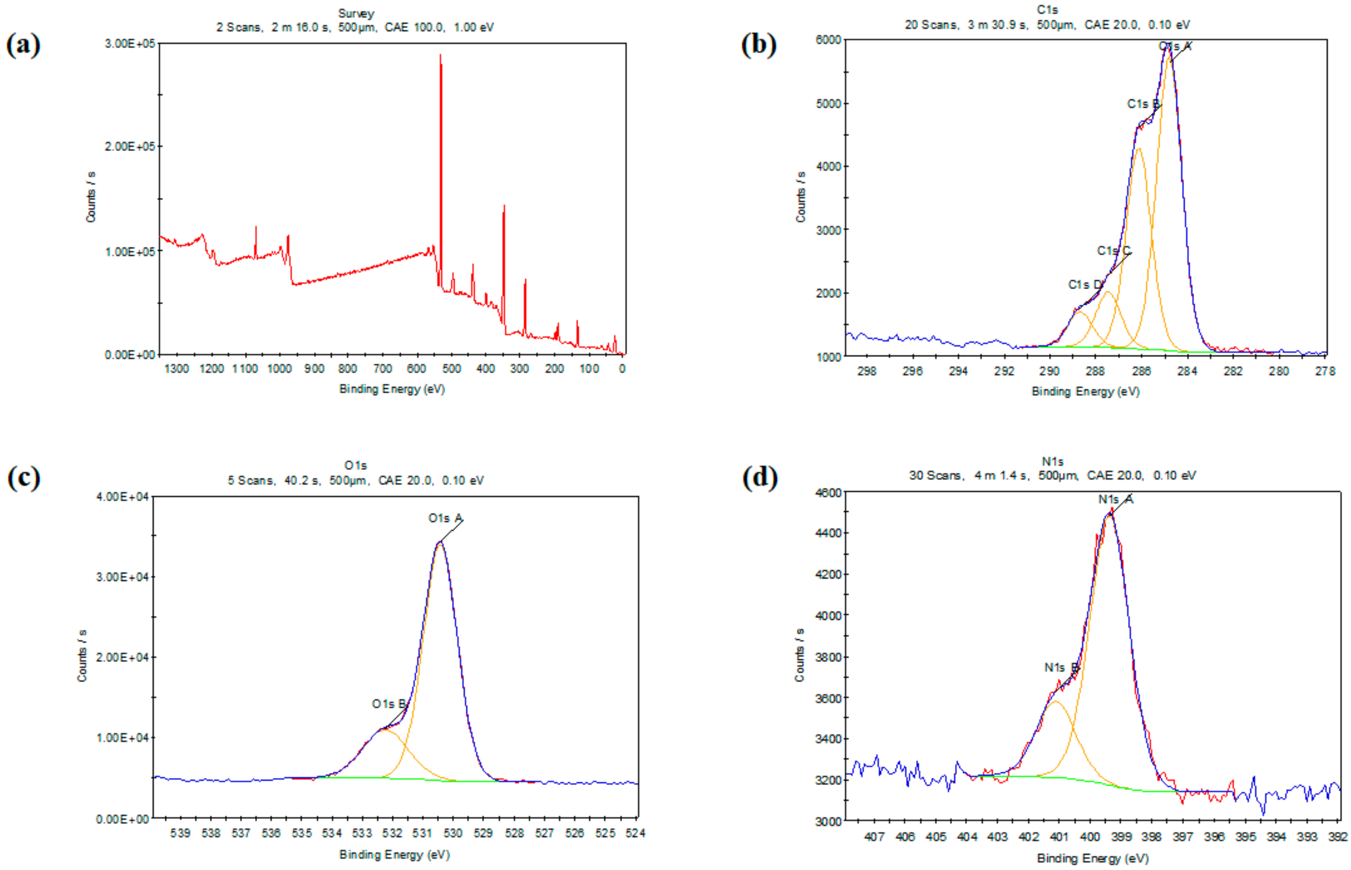

Figure S3. (a) XPS spectrum and peak-differentiation-imitating analysis of (b) C1s, (c) O1s, and (d) N1s species of HA 500p

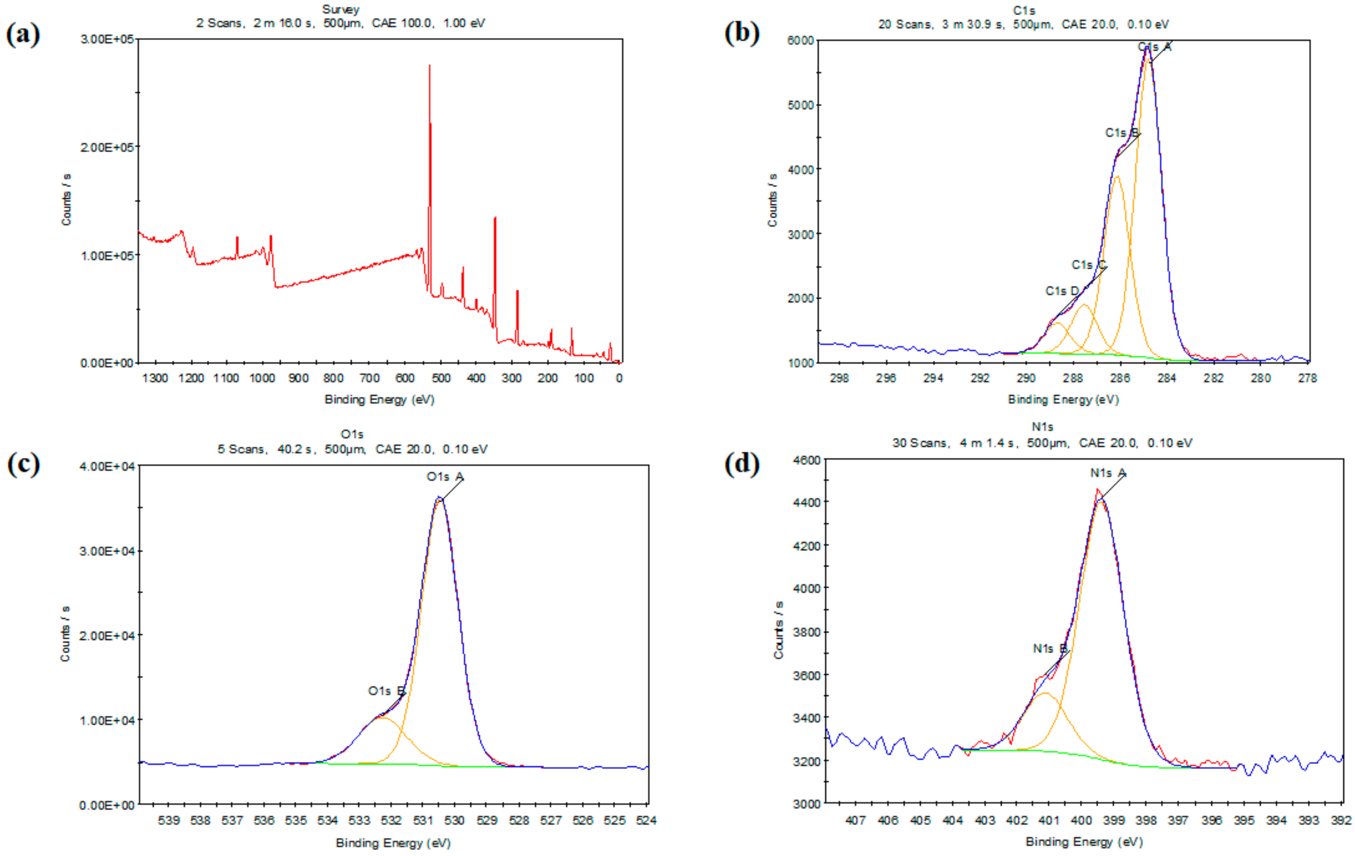

Figure S4. (a) XPS spectrum and peak-differentiation-imitating analysis of (b) C1s, (c) O1s, and (d) N1s species of HA 1000p

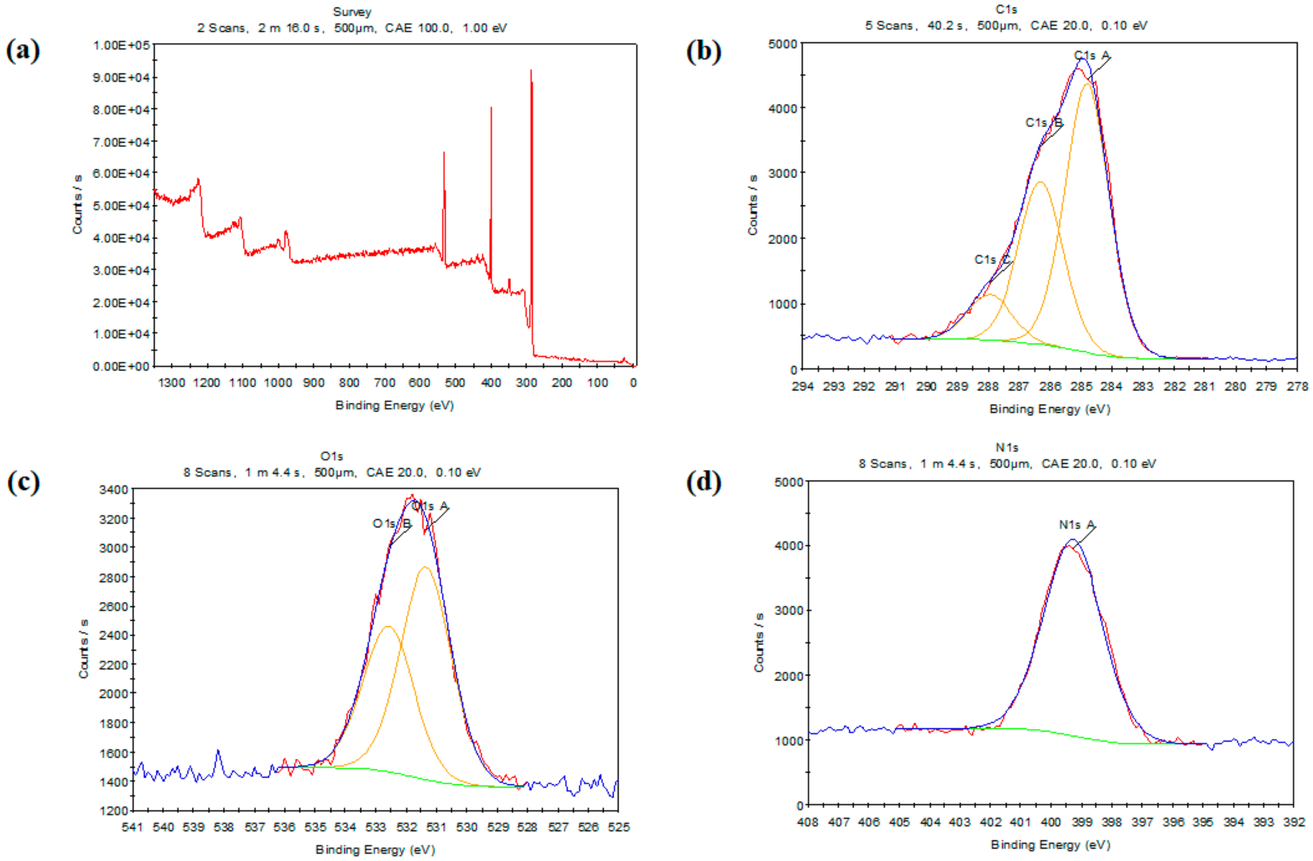

Figure S5. (a) XPS spectrum and peak-differentiation-imitating analysis of (b) C1s, (c) O1s, and (d) N1s species of PIIID-HA discs

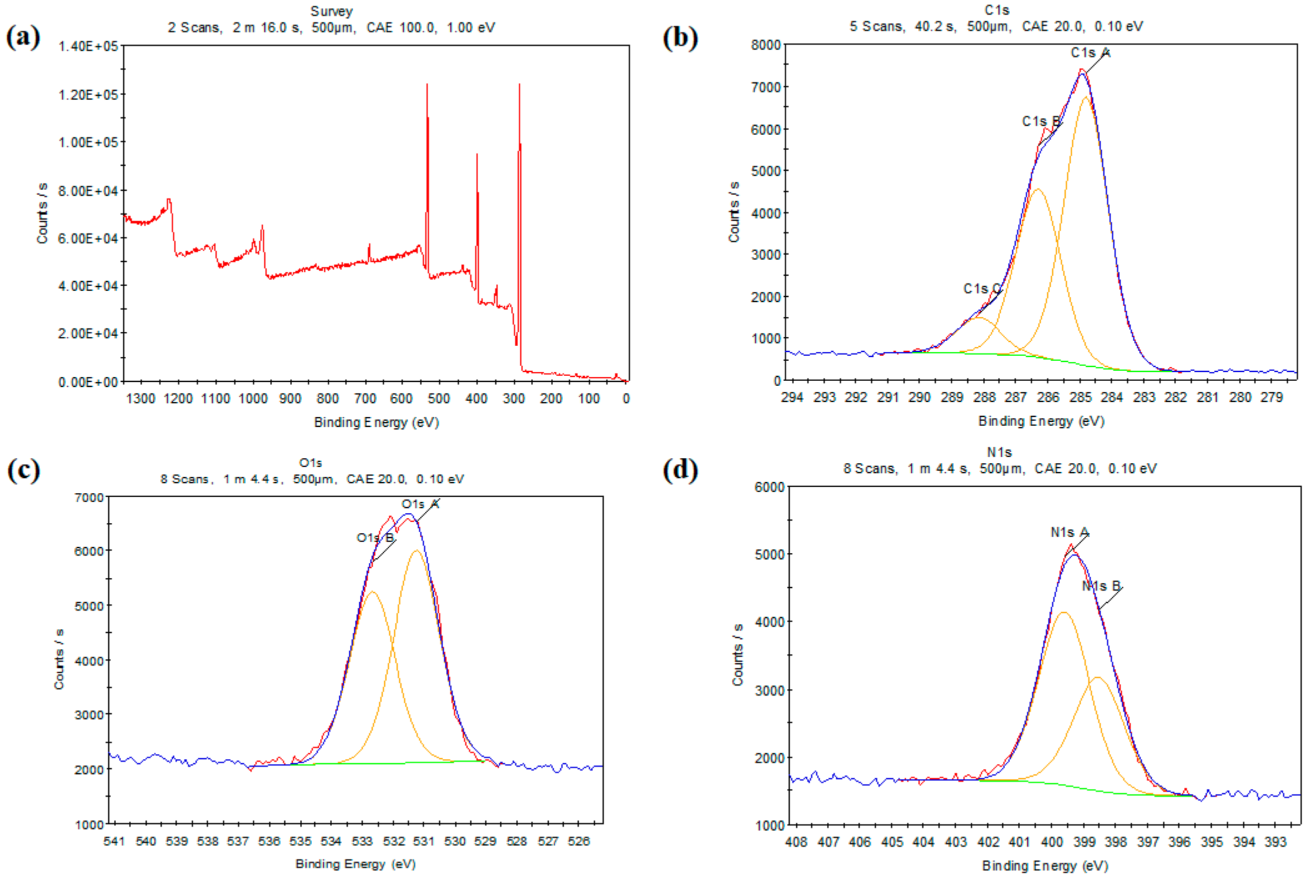

Figure S6. (a) XPS spectrum and peak-differentiation-imitating analysis of (b) C1s, (c) O1s, and (d) N1s species of PIIID-HA 500p

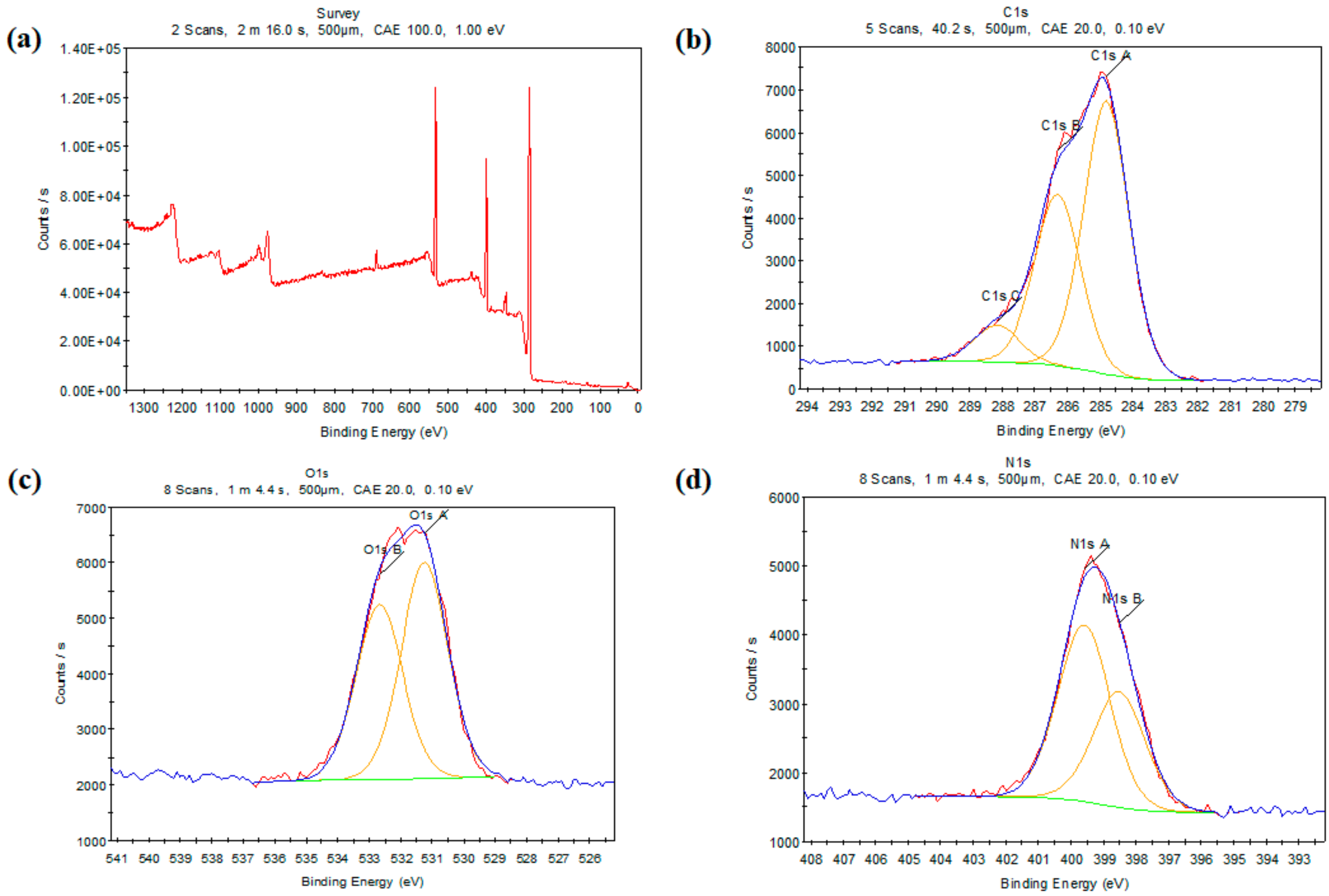

Figure S7. (a) XPS spectrum and peak-differentiation-imitating analysis of (b) C1s, (c) O1s, and (d) N1s species of PIIID-HA 1000p
